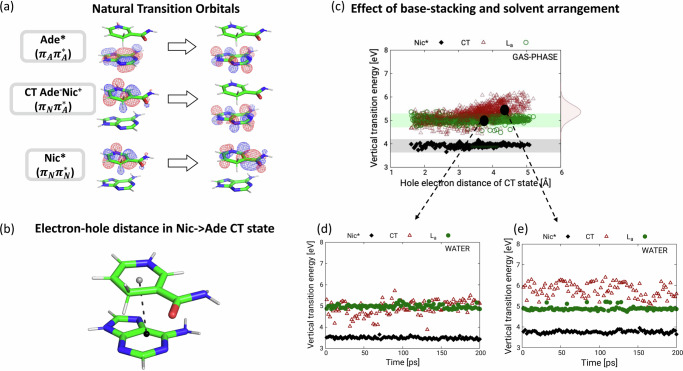# Author Correction: Sub-100-fs energy transfer in coenzyme NADH is a coherent process assisted by a charge-transfer state

**DOI:** 10.1038/s41467-024-52280-y

**Published:** 2024-09-10

**Authors:** Vishal Kumar Jaiswal, Daniel Aranda Ruiz, Vasilis Petropoulos, Piotr Kabaciński, Francesco Montorsi, Lorenzo Uboldi, Simone Ugolini, Shaul Mukamel, Giulio Cerullo, Marco Garavelli, Fabrizio Santoro, Artur Nenov

**Affiliations:** 1 Dipartimento di Chimica industriale “Toso Montanari”, Università di Bologna, Viale del Risorgimento 4, 40136 Bologna, Italy; 2 ICMol, Universidad de Valencia, Catedrático José Beltrán Martínez, 2, 46980 Paterna, Spain; 3 Dipartimento di Fisica, Politecnico di Milano, Piazza Leonardo da Vinci 32, 20133 Milano, Italy; 4 Department of Chemistry and Department of Physics and Astronomy, University of California, Irvine, CA 92697 USA; 5 Istituto di Chimica dei Composti Organometallici (ICCOM-CNR), Area della Ricerca del CNR, Via Moruzzi 1, I-56124 Pisa, Italy

**Keywords:** Excited states, Energy transfer, Quantum chemistry, Optical spectroscopy

Correction to: *Nature Communications* 10.1038/s41467-024-48871-4, published online 08 June 2024

The original Article contained an error in Fig. 3e, in which the data points for the CT states were incorrectly reported as green full dots and labelled as L_a_, and the data points for the L_a_ state were incorrectly reported as red empty triangles and labelled as CT. In the corrected version, the data points of the CT state are shown as red empty triangles and the data points for the L state are shown as green full dots. This has been corrected in both the PDF and HTML versions of the Article.

CORRECTED FIGURE