# Supplementary material for: Sub-100-fs energy transfer in coenzyme NADH is a coherent process assisted by a charge-transfer state
Source: Nat Commun. 2024 Jun 8;15:4900. doi: 10.1038/s41467-024-48871-4 (PMC11162464; doi:10.1038/s41467-024-48871-4)
Supplement: Supplementary file 1 — Supplementary information [file 41467_2024_48871_MOESM1_ESM.pdf]

Supporting Information

Sub-100-fs energy transfer in coenzyme NADH is a coherent process assisted by a charge-transfer state.

Vishal Kumar Jaiswal[1], Daniel Aranda Ruiz[2‡], Vasilis Petropoulos[3‡], Piotr Kabaciński[3‡], Francesco Montorsi[1], Lorenzo Uboldi[3], Simone Ugolini[1], Shaul Mukamel[4], Giulio Cerullo[3,*], Marco Garavelli[1,*], Fabrizio Santoro[5], Artur Nenov[1,*]

[1]Dipartimento di Chimica industriale "Toso Montanari", Università di Bologna, Viale del Risorgimento 4, 40136 Bologna, Italy;

[2]ICMol, Universidad de Valencia, Catedrático José Beltrán Martínez, 2, 46980 Paterna, Spain;

[3]Dipartimento di Fisica, Politecnico di Milano, Piazza Leonardo da Vinci 32, 20133 Milano, Italy;

[4]Department of Chemistry and Department of Physics and Astronomy, University of California, Irvine, California 92697, United States;

[5]Istituto di Chimica dei Composti Organometallici (ICCOM-CNR), Area della Ricerca del CNR, Via Moruzzi 1, I-56124 Pisa, Italy;

‡ *These authors have equally contributed to this work*

* *Corresponding authors: artur.nenov@unibo.it, marco.garavelli@unibo.it, giulio.cerullo@polimi.it*

Table of Contents

SUPPL. NOTE. 1 TRANSIENT ABSORPTION EXPERIMENT ... 3

1.1 TA MAP OF UV-EXCITED NADH IN METHANOL ... 3
1.2 GLOBAL FITTING OF UV-EXCITED ADENOSINE AND NADH DYNAMICS ... 3
1.3 ESTIMATION OF THE ENERGY TRANSFER TIMESCALE WITHIN THE NADH DIMER IN WATER SOLVENT (ADENINE TO NICOTINAMIDE ENERGY TRANSFER) ... 6

SUPPL. NOTE. 2 REPLICA EXCHANGE MOLECULAR DYNAMICS ... 8

2.1 MODEL SETUP ... 8
2.2 PRE-EQUILIBRATION AT 298.15 K ... 8
2.3 EQUILIBRATION AT REPLICA TEMPERATURE ... 8
2.4 PRODUCTION (REMD) ... 8

SUPPL. NOTE. 3 CLUSTER ANALYSIS ... 9

SUPPL. NOTE. 4 QM/MM SETUP AND PROTOCOL FOR OBTAINING LVC PARAMETERS. ... 10

SUPPL. NOTE. 5 BENCHMARKING OF CASSCF ACTIVE-SPACE ... 12

SUPPL. NOTE. 6 VERTICAL TRANSITION ENERGIES ON CLUSTER REPRESENTATIVES ... 14

SUPPL. NOTE. 7 DIABATIZATION OF LE AND CT STATES ... 15

7.1 Electronic Coupling (V), Energy-gap (ΔE) and Mixing (V / ΔE) values for 100 different solute conformations of most populated cluster C0. 17
7.2 Adiabatic and diabatic states of frozen solute in 100 solvent ensemble. 17

SUPPL. NOTE. 8 WAVEPACKET DYNAMICS ON CLUSTER REPRESENTATIVES 19

8.1 LVC parameters for dynamics on Cluster representatives in diabatic basis 19
8.2 Wavepacket dynamics for CT-mediated EET including all coupling terms in vibronic Hamiltonian in diabatic-basis. 20
8.3 Wavepacket dynamics for Direct EET including all coupling terms in vibronic Hamiltonian in diabatic-basis. 22

SUPPL. NOTE. 9 ENERGIES OF DIABATIC STATES ALONG EXAMPLE ML-MCTDH DYNAMICS.1. 24

SUPPL. NOTE. 10 SELECTIVE EFFECTS OF INTERSTATE ELECTRONIC/VIBRONIC COUPLING ON WAVEPACKET DYNAMICS. 25

SUPPL. NOTE. 11 SELECTIVE EFFECT OF REMOVING LOW-FREQUENCY MODES ON WAVEPACKET DYNAMICS 44

SUPPL. NOTE. 12 PHOTOACTIVE NORMAL MODES RESPONSIBLE FOR EET TRANSFER 45

SUPPL. NOTE. 13 BRANCHING-SPACE FOR CT-MEDIATED AND DIRECT MECHANISM 46

SUPPL. NOTE. 14 WAVEPACKET DYNAMICS ON FROZEN SOLUTE IN SOLVENT ENSEMBLE 47

14.1 Generation of solvent ensemble 47
14.2 Wavepacket dynamics on solvent ensemble in absence of CT-state 47

SUPPL. NOTE. 15 COHERENCES BETWEEN THE ELECTRONIC STATES ALONG EET DYNAMICS FOR NADH . 48

Suppl. Note. 1 Transient Absorption Experiment

1.1 TA map of UV-excited NADH in methanol

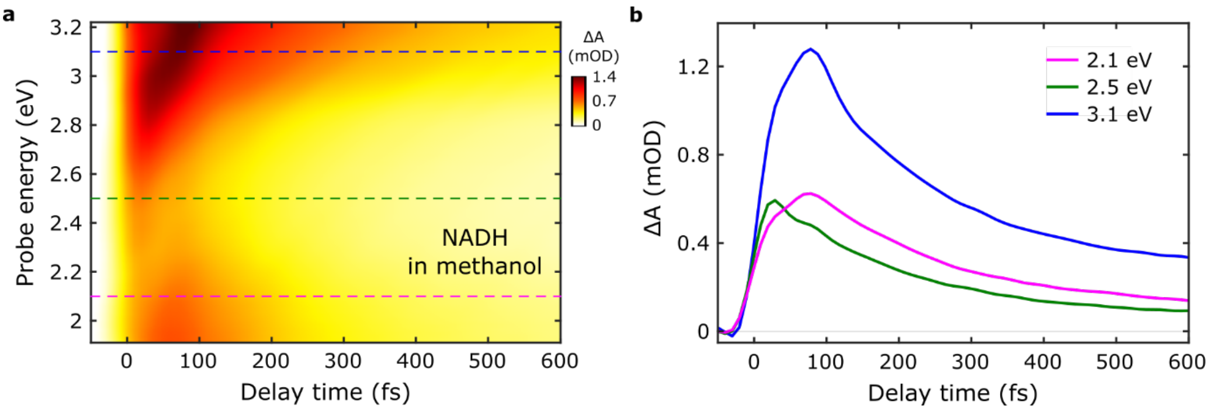

Suppl. Fig. 1: NADH in methanol upon 4.7 eV excitation, focusing the early time evolution. (a) TA map as a function of delay time and probe photon energy; (b) TA dynamics at selected probe photon energies, as highlighted in (a).

1.2 Global Fitting of UV-excited Adenosine and NADH dynamics

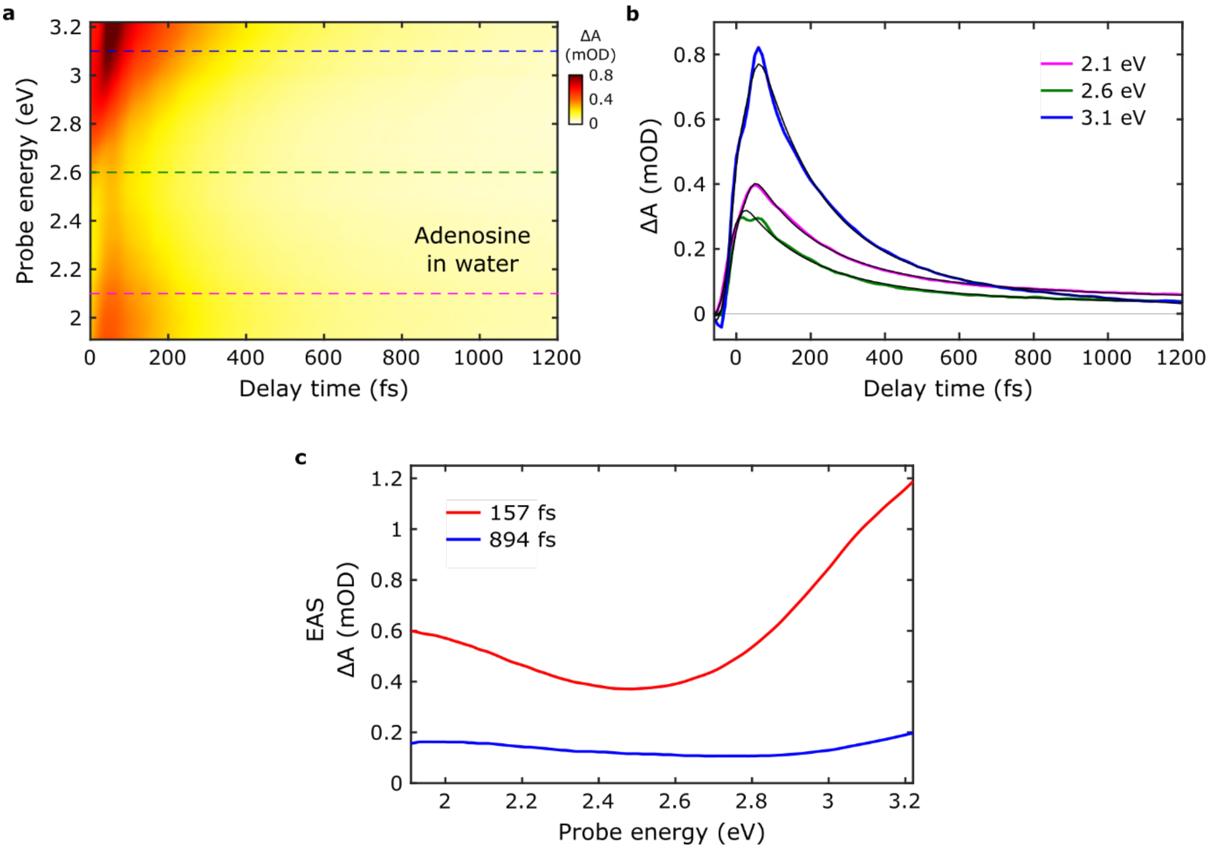

Suppl. Fig. 2: Global fitting results of adenosine in water upon 4.7 eV excitation. (a) TA map as a function of delay time and probe photon energy; (b) TA dynamics at specific probe photon energies, as highlighted in (a), overlapped with their fit (black line) extracted by global analysis. (c) Evolution

associated spectra (EAS) obtained from the global analysis of the TA data, with the corresponding time constants.

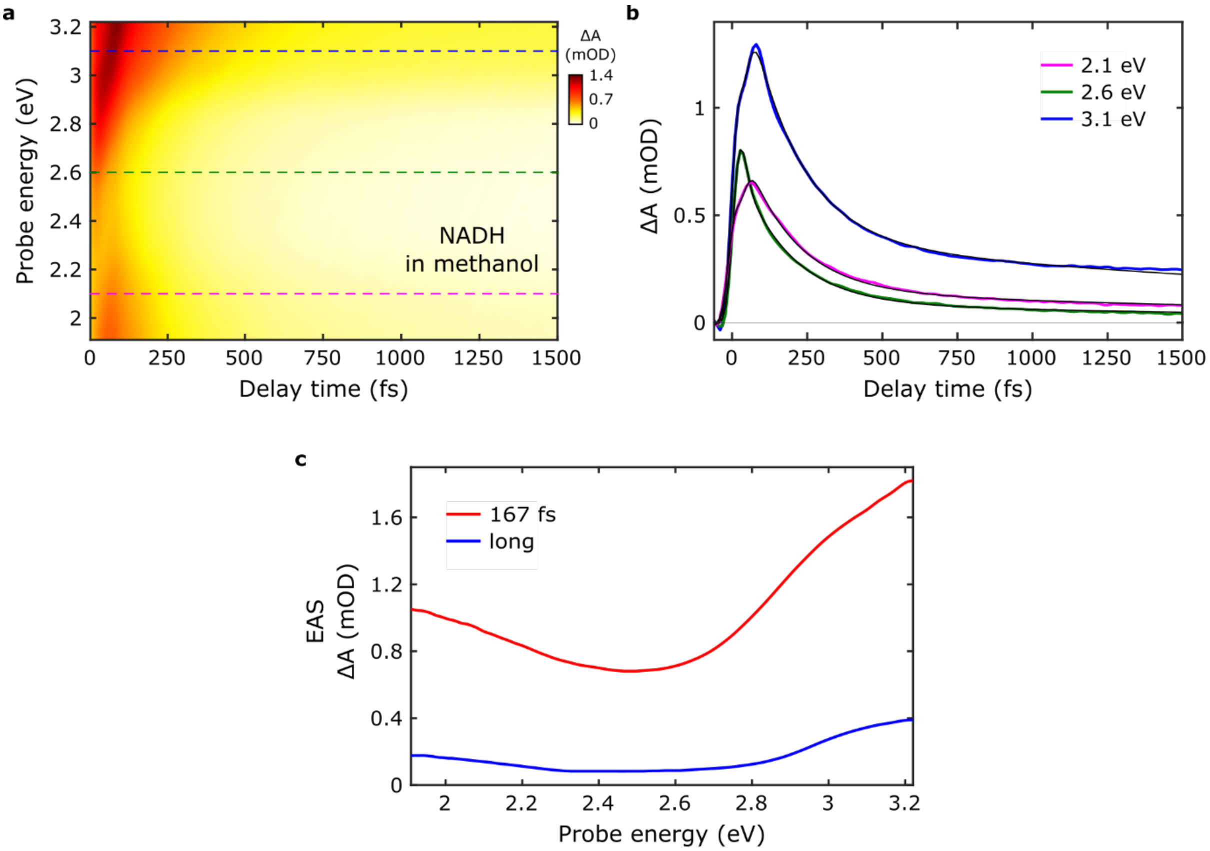

Suppl. Fig. 3: Global fitting results of NADH in methanol upon 4.7 eV excitation. (a) TA map as a function of delay time and probe photon energy; (b) Selected dynamics at specific probe photon energies, as highlighted in (a), overlapped with their fit (black line) extracted by global analysis. (c) EAS spectra obtained from the global analysis of the TA data, with the corresponding time constants.

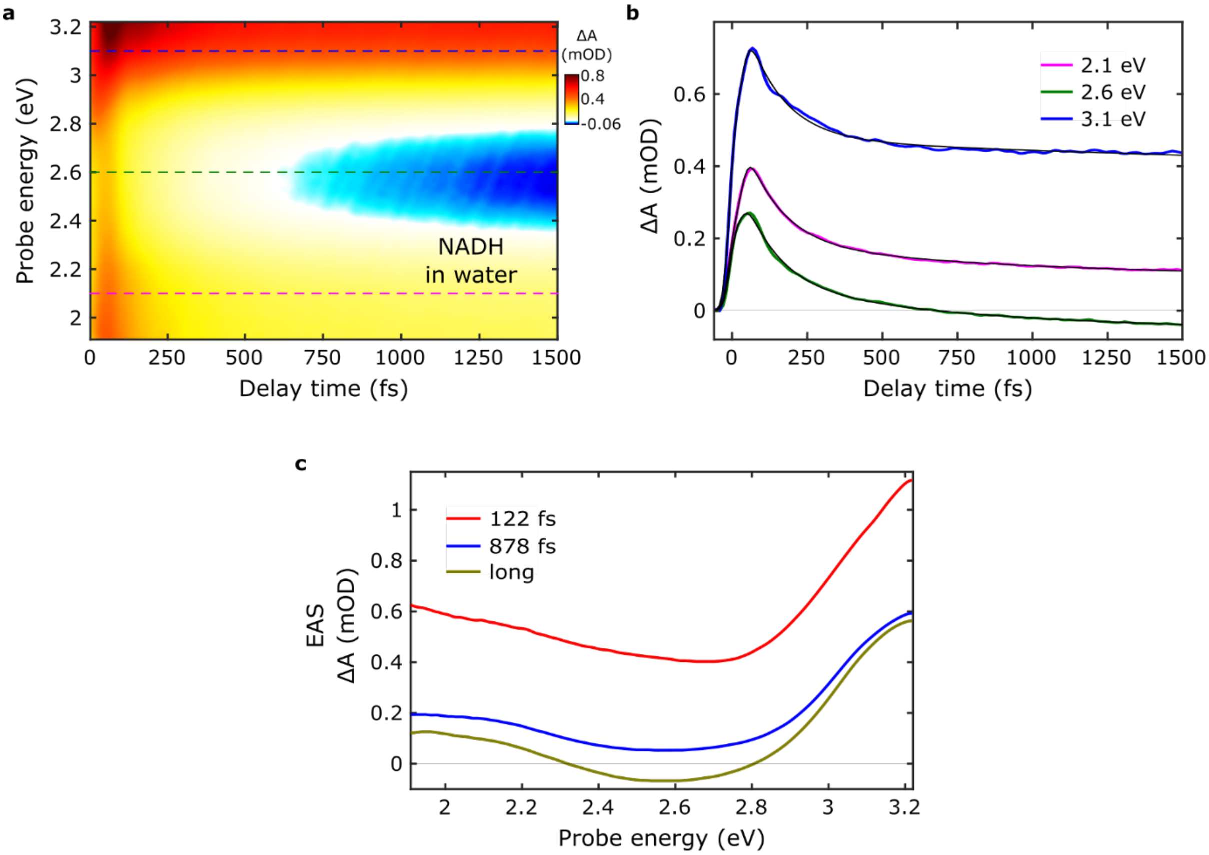

Suppl. Fig. 4: Global fitting results of NADH in water upon 4.7 eV excitation. (a) TA map as a function of delay time and probe photon energy; (b) TA dynamics at selected probe photon energies, as highlighted in (a), overlapped with their fit (black line) extracted by global analysis. (c) EAS spectral evolution obtained from the global analysis of the TA data, with the corresponding time constants.

	τ_1 (fs)	τ_2 (fs)	τ_3 (fs)
**Adenosine in water**	157 ± 4	894 ± 50	-
**NADH in methanol**	167 ± 4	-	long
**NADH in water**	122 ± 4	878 ± 40	long

Suppl. Table 1: Time constants, accompanied by their experimental uncertainty, as obtained from the global fitting of the TA data in case of Adenosine in water, NADH in methanol and NADH in water.

1.3 Estimation of the energy transfer timescale within the NADH dimer in water solvent (Adenine to Nicotinamide energy transfer)

NADH dissolved in water exists in an equilibrium between the folded and unfolded conformers. In such conditions, the folded conformers constitute approximately the 30% population and play a vital role in bridging the Adenine (Ade) and Nicotinamide (Nic) moieties, thereby enabling the energy transfer from Ade La-state to Nicotinamide excited state (Nic*). As mentioned in the main text, upon photoexcitation of NADH in water at 4.7 eV, two possible excited state pathways emerge (Scheme 1):

(i) Photoexcitation of the unfolded NADH conformers (70%). Taking into account the established negligible energy transfer in the open configuration[1]the ultrafast kinetics of the NADH unfolded conformers should retain a similar depopulation rate as Ade in water ($\tau_{\text{NADH, unfolded}} \approx \tau_{1\text{Ade, water}}$). The experimentally resolved ultrafast component of Adenosine's excited state in water (Suppl. Table 1), $\tau_{1\text{Ade, water}}$ = 157 fs, corresponds to the main depopulation channel via internal conversion (IC) back to the ground state. Indeed, in our experiment for NADH dissolved in methanol, where NADH exists in open configuration, the Ade La-state possess a similar ultrafast component $\tau_{1\text{NADH, methanol}}$ = 167 fs. The slight deviation in the latter lifetime with respect to the monomeric Ade in water can be attributed to the different solvent environment.

(ii) Photoexcitation of the folded NADH conformers (30%). In folded conformers, the excited state energy faces two concurrent dissipation channels: (a) Ade* IC back to the ground state and (b) ultrafast energy transfer from Ade* to Nic*. The competition between these two ultrafast processes is manifested in the TA measurements as an increase in the overall depopulation rate of the Ade La-state. We note that our pump pulse resonantly excites the Ade component in NADH dimer. Since the Ade depopulation is way faster than any other excited state relaxation events (e.g., vibrational cooling of Nic* takes place in picosecond timescales[1,2], we can safely assign the differences found between the τ_1 components of Adenosine and NADH in water solvent (Suppl. Table 1) to the presence of additional deactivation channels in Ade*. Indeed, for NADH in water we obtain $\tau_{1\text{NADH, water}}$ = 122 fs from global analysis (Suppl. Table 1), a component faster than the net depopulation lifetime of the monomeric Ade* $\tau_{1\text{Ade, water}}$ = 157 fs.

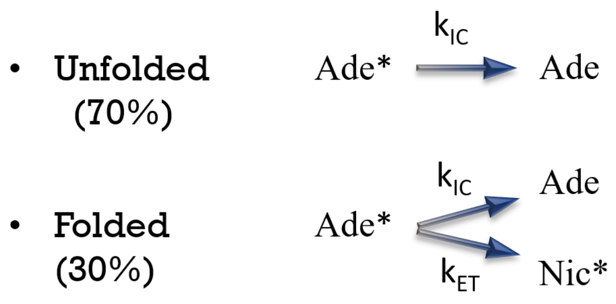

Scheme 1: Possible kinetic pathways of NADH in water following resonant Ade photoexcitation.

Thus, taking into account the 30/70 population ratio between folded/unfolded conformers of NADH in water, the $\tau_{1\text{NADH, water}}$ = 122 fs lifetime extracted by global analysis can be considered as a weighted average of the folded and unfolded ultrafast lifetime components, simply given by:[3]

$$\tau_{1\,NADH,\,water} = 0.7\,\tau_{NADH,\,unfolded} + 0.3\,\tau_{NADH,\,folded} \qquad \text{(Eq. 1)}$$

As we mentioned earlier, we can safely assign $\tau_{NADH,\,unfolded} = \tau_{1\,Ade,\,water} = 157$ fs in Eq. 1, resulting in:

$$\tau_{NADH,\,folded} = 40 \pm 6 \text{ fs} \qquad \text{(Eq. 2)}$$

Accompanied by a value uncertainty retrieved through simple error propagation from the values of Suppl. Table 1.

The total decay rate of the folded NADH components ($\mathbf{k}_{total}$ = (40 fs)$^{-1}$) reflects the sum of the Ade internal conversion ($\mathbf{k}_{IC}$= (157 fs)$^{-1}$) and the ultrafast energy transfer from Ade La-state to Nic* ($\mathbf{k}_{ET}$), given simply as:[1,3]

$$\mathbf{k}_{total} = \mathbf{k}_{IC} + \mathbf{k}_{ET} \qquad \text{(Eq. 3)}$$

Resulting in an energy transfer from Ade La-state to Nic*:

$$\tau_{ET,\,folded} = 54 \pm 11 \text{ fs} \qquad \text{(Eq. 4)}$$

However, we should stress the 4.7 eV resonant excitation of the Ade moiety, might result in concurrent direct population of a small percentage of the Nic moiety. Taking into account that: (i) the absorption of Nic at 4.7 eV is ≈12 times lower in amplitude than its absorption peak around 3.6 eV [3,4](ii) the extinction coefficient of Ade in water at 4.7 eV (≈13000 cm^{-1} M^{-1})[5] (iii) the extinction coefficient of NADH in water at 3.6 eV (≈6300 cm^{-1} M^{-1})[6,7] ; the anticipated direct excitation of the Nic moiety is expected to be around 1/24 of the contribution of the Ade absorption at 4.7 eV. As a result, the 2 OD absorption at 4.7 eV in our experiment corresponds to 1.92 OD of Ade and 0.08 OD of Nic absorptions, respectively.

Despite the 4% of direct Nic population upon 4.7 eV excitation, the vibrational relaxation to the Nic minimum occurs in mere picoseconds[1,2] , at least one order of magnitude slower than the EET itself. This process may only influence the longer time constants of our global analysis, namely, the τ_2 and τ_3 components of Suppl. Table 1. Thus, the τ_1 time constant of NADH in water exclusively illustrates the additional deactivation channel found in Ade due to the presence of the EET, being unaffected by the small percentage (4%) of Nic direct excitation and without influencing the result of our master equation Eq.1.

Suppl. Note. 2 Replica Exchange Molecular Dynamics

The replica exchange dynamics were run using the protocol described for the simulation of dinucleosides.[8] The REMD simulations were done with integration time-steps of 4fs using hydrogen mass repartitioning (HMR) as outlined in the reference dynamical study. In total 20 ns of REMD were performed covering a temperature of 280 to 396 K over 18 replicas. The dynamics were post processed by extracting trajectories at 298 K.

2.1 Model Setup

The setup for MD consisted of NADH solvated in a truncated octahedral box of TIP3P water with an 8 Angstrom buffer space and then charge neutralized with 2 sodium ions. The parametrized forcefield parameters for NADH were taken from previously published works.[9,10] 18 replicas were setup with temperatures set at 280.00, 286.04, 292.18, 298.41, 304.74, 311.17, 317.69, 324.31, 331.03, 337.85, 344.79, 351.82, 358.97, 366.22, 373.59, 381.07, 388.67, and 396.40.

2.2 Pre-Equilibration at 298.15 K

All replicas were optimized with 1000 steps of steepest descent with 5 kcal/(mol·Å^2) positional restraints on NADH heavy atoms, followed by 15 ps of MD with same positional restraints at NVT ensemble. This was followed 3 optimizations of 1000 steps each with steepest descent with relaxing positional restraints of 2,0.1 and 0 kcal/(mol·Å^2) on NADH heavy atoms. Then a series of MD with relaxing positional restraints on heavy atoms were performed. 5ps of MD was done with 1 kcal/(mol·Å^2) positional restraints on NADH heavy atoms. This was followed by 1ns MD with 0.5 kcal restraints on NADH heavy atoms, then 1ns of MD with 0.5 kcal restraints on NADH backbone heavy atoms and then 1ns MD with no positional restraints. These series of dynamics were all done with integration timestep of 1fs except the last unconstrained MD where a timestep of 2fs was used. All MD steps were done by keeping the temperature at 298.15 K using the weak-coupling algorithm with 1ps time constant and using SHAKE to constrain the bonds involving hydrogen atoms. All MD simulations were done at NVT ensemble with periodic boundary conditions and PME with 8 Angstrom cutoff.

2.3 Equilibration at Replica temperature

The previous step results in 18 replicas at 298.15 K. For final equilibration step before running REMD, each replica was equilibrated from 298.15 K to the respective replica temperature (from 280 to 396 K) over 1ns. A Langevin thermostat with 2ps^{-1} collision frequency was employed. HMR was used to enable timestep of 4fs. The last structure from these dynamics were used to initiate REMD simulations.

2.4 Production (REMD)

REMD simulations were done for 18 replicas at temperatures mentioned above with 1ps exchange attempt interval. Dynamics were run with integration step of 4fs and HMR. The temperature was maintained by a Langevin thermostat with 2ps^{-1} collision frequency. All simulations were done at NVT ensemble with periodic boundary conditions and PME with 8 Angstrom cutoff. Simulations were performed for 20ns.

Suppl. Note. 3 Cluster Analysis

The dynamics were post processed by extracting trajectories at 298 K. which resulted in 2000 MD frames on which subsequent clustering was done using cpptraj program from AmberTools. Clustering was performed using DBSCAN[11] algorithm with an epsilon of 0.6, using the distance matrix of the solute atoms as a metric.

**CLUSTERS from distance-matrix (DME) clustering // threshold = 0.6**	**Percentage population in total REMD dynamics.**	**Median Interbase distance (ANGSTROM)**
Cluster C0	21.8	4.12
Cluster C1	13.1	4.73
Cluster C2	10.2	4.60
Cluster C3	6.1	4.18
Cluster C4	3.4	5.86
Cluster C5	2.3	4.48
Cluster C6	2.3	3.85
Cluster C7	0.9	8.45

Suppl. Table 2: Cluster population and mean interbase distance in Angstroms of various clusters from REMD dynamics

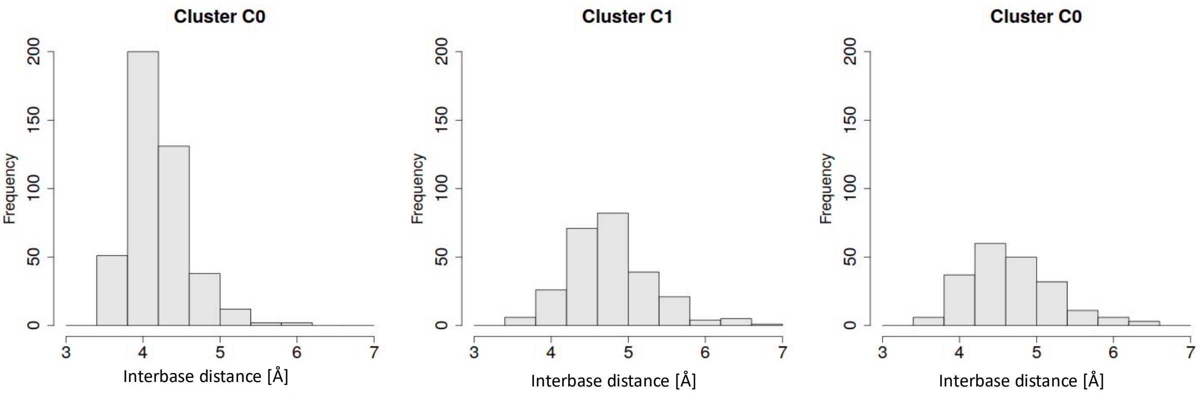

Suppl. Fig. 5 Distribution of inter-base distance in Angstroms for three largest clusters

Suppl. Note. 4 QM/MM Setup and Protocol for obtaining LVC parameters.

A QM/MM setup of a spherical droplet was made from the snapshots selected to enable ab-initio computations with electrostatic embedding. The spherical droplet consisted of 500 closest solvent molecules from the center of geometry of the solute. A High/Medium/Low layer partitioning was used where the high layer is described quantum mechanically and the medium and low layers through molecular mechanics force-fields. The medium layer is mobile during computations, while the low-layer is kept frozen. The high layer consisted of the two bases. The rest of the solute consisting of the backbone and nearest hydrogen-bonded waters were kept in the mobile medium layer, and the rest of the surrounding solvents were kept frozen in low-layer.

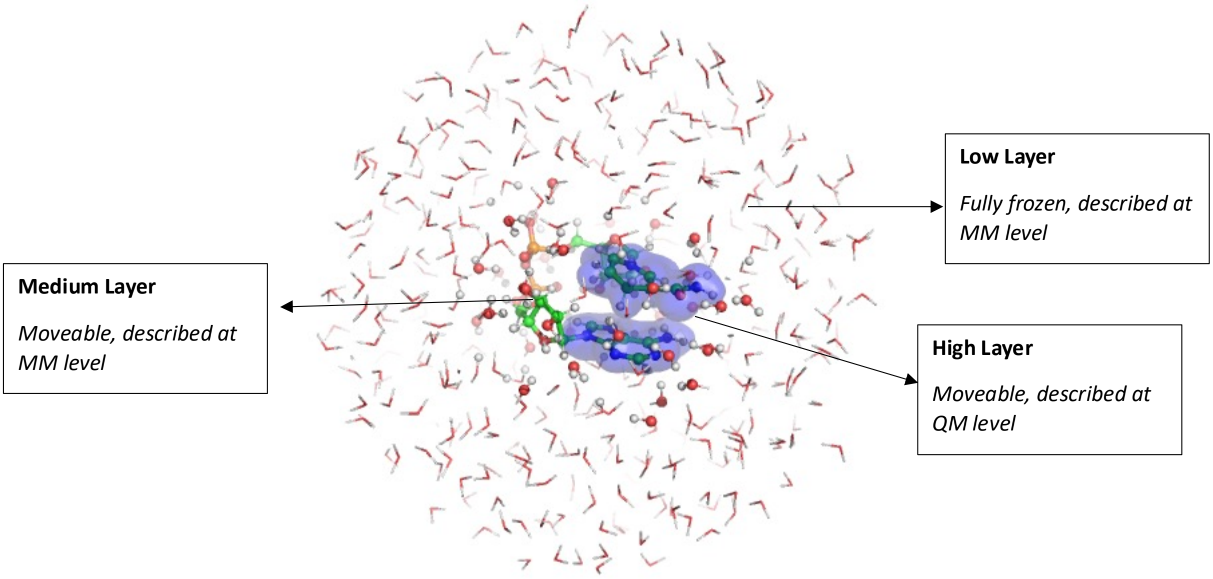

Suppl. Fig. 6 QM/MM setup used for optimizations where both High and Medium layer are moveable while Low layer is kept frozen. For energy and gradient computations to build the LVC model, only the high layer described at QM-level was movable, rest comprising backbone and solvent were kept frozen. The two chromophores are in High layer. The backbone of the nucleotide along with nearest water molecules shown with balls and sticks are in Medium layer. Rest of the water molecules shown with lines are in Low layer.

Using this QM/MM setup the ground-state was optimized at MP2 level of theory using 6-31G* basis-set. Post-optimization a frequency computation was done to obtain the normal-modes of the system. Only the frequency of the High-layer was computed owing to the ultrafast nature of the EET process. The frequency computations were done on every snapshot for which ML-MCTDH dynamics were performed to obtain system-specific normal modes accounting the effect of solute structure and environment. These system-specific normal modes were used to generate displaced structures on which electronic structure computations were done to obtain parameters for LVC Hamiltonian.

Suppl. Note. 5 Benchmarking of CASSCF active-space

To enable multiconfigurational excited-state computations, a selection of active-space orbitals is needed. The valence π-system of NADH consists of a large number of orbitals which have to be truncated to enable computations in reasonable time. To enable this, benchmark computations were done with full active-space using the RASPT2 methodology by putting all 15 occupied orbitals in RAS1 and 7 virtual orbitals in RAS3 and allowing 4 excitations. These benchmark computation on the stacked structure reveal a dark Nic->Ade charge transfer state between the bright La and Nic* state.

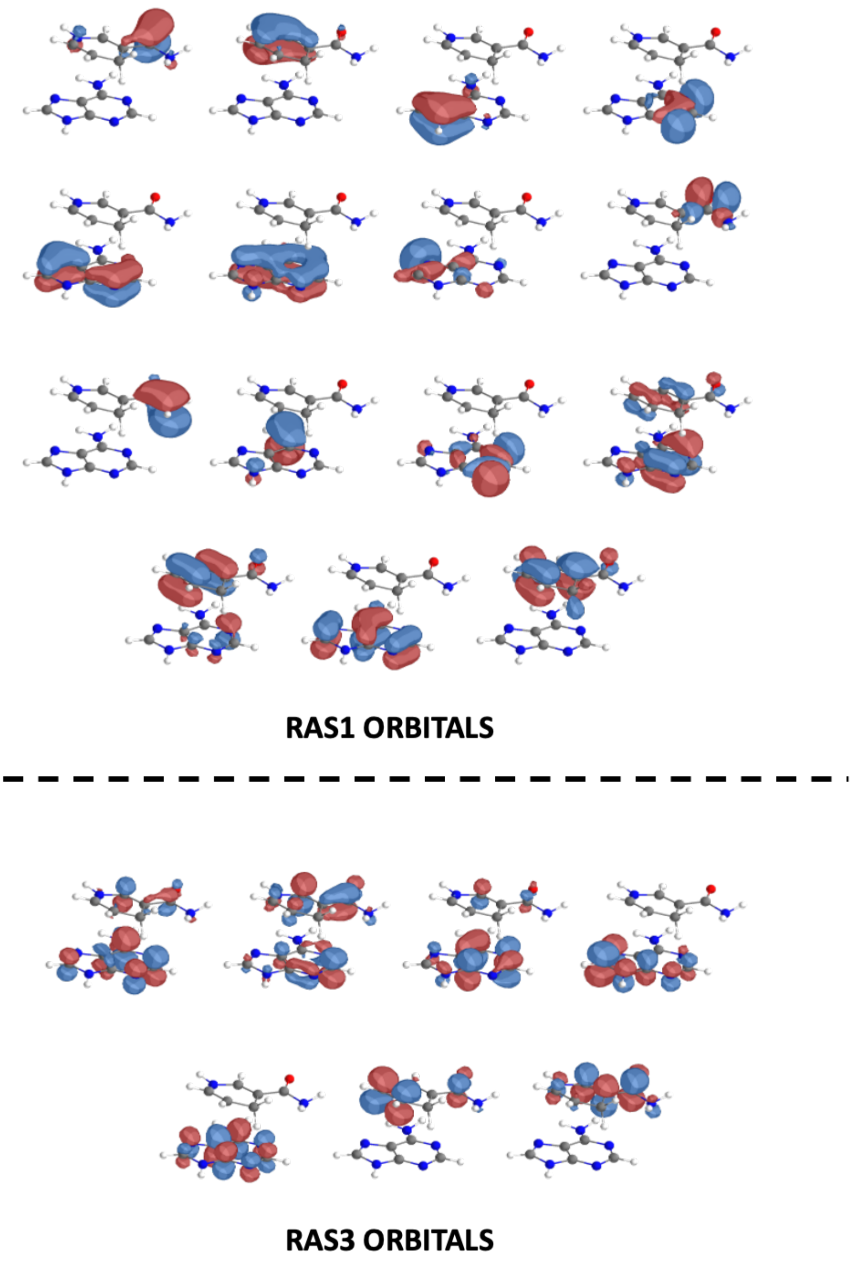

Suppl. Fig. 7 Active-Space orbitals of 4,15||4,7 RASPT2 computation

Natural Transition Orbital analysis reveal all the low-lying excitations relevant for ultrafast EET process to be described mainly by single transitions. Therefore, a reduced active space of |4,4| (4 electrons in 4 orbitals) was chosen to be a reasonable approximation. To enable smooth potentials at CASPT2 level, the XMS-CASPT2 level of theory was employed. A comparison of the vertical transitions for the chosen stacked snapshot with different active spaces are shown in Table 2. The level of underlying RAS/CAS theory is specified in the tables in the standard way as described below:

The various RAS subspaces are denoted as **RAS 1 | RAS2 | RAS3 ,**

a,b | c,d | e,f is a RASSCF computations with **b** orbitals in RAS1 space, **d** orbitals in RAS2 space and **f** orbital sin RAS3 space. There are **c** electrons in RAS2 space. A maximum of **a** excitations from RAS1 space and **e** excitations into RAS3 space is allowed.

	**4,15||4,7**	**|14,14|**	**|12,12|**	**|10,10|**	**|8,8|**	**|6,6|**	**|4,4|**
**SSPT2**	eV	eV	eV	eV	eV	eV	eV
Nic*	3.48	3.32	3.33	3.33	3.45	3.23	3.26
CT	4.76	5.37	5.45	5.54	4.87	4.76	4.77
La	5.05	5.15	5.27	5.25	5.04	5.05	4.93

Suppl. Table 3: Vertical Excitation energies at SS-CASPT2 level for various active spaces.

	**SSPT2**				**XMSPT2**		
	**4,15||4,7**	**|8,8|**	**|6,6|**	**|4,4|**	**|8,8|**	**|6,6|**	**|4,4|**
**SSPT2**	**eV**	eV	eV	eV	eV	eV	**eV**
Nic*	**3.48**	3.45	3.23	3.26	3.44	3.36	**3.40**
CT	**4.76**	4.87	4.76	4.77	4.92	4.86	**4.83**
La	**5.05**	5.04	5.05	4.93	5.14	5.00	**4.99**

Suppl. Table 4: Comparison between Vertical Excitation energies at SS-CASPT2 vs XMS-CASPT2 level for various active spaces. Note that XMS-CASPT2 with **|4,4|** active-space gives values comparable to the **reference 4,15||4,7** computation. Therefore, XMS-CASPT2 with a minimal |4,4| active-space was utilized in computations for gathering parameters for LVC Hamiltonian.

Suppl. Note. 6 Vertical Transition energies on cluster representatives

REP C0	eV (*f*)										AVG (eV)	σ (eV)
Nic*	3,61 (0.13)	3,48 (0.13)	3,82 (0.11)	3,59 (0.12)	3,57 (0.13)	3,74 (0.13)	3,60 (0.14)	3,74 (0.11)	3,43 (0.14)	3,63 (0.13)	3.62	0.12
CT	5,30 (0.03)	4,95 (0.18)	4,64 (0.00)	4,76 (0.00)	5,15 (0.09)	4,96 (0.00)	4,74 (0.00)	4,77 (0.02)	3,79 (0.00)	5,16 (0.02)	4.82	0.42
LA	5,07 (0.56)	5,04 (0.42)	5,00 (0.60)	5,10 (0.59)	4,99 (0.52)	5,44 (0.60)	4,98 (0.60)	5,02 (0.57)	4,89 (0.59)	5,04 (0.58)	5.06	0.15
LA-CT gap	-0,23	0,09	0,36	0,34	-0,16	0,48	0,24	0,25	1,10	-0,12		
REP C1												
Nic*	3,83 (0.12)	3,98 (0.10)	3,71 (0.16)	3,93 (0.09)	3,67 (0.13)	3,73 (0.12)	4,02 (0.10)	3,88 (0.12)	3,91 (0.12)	4,11 (0.09)	3.88	0.14
CT	5,20 (0.18)	4,52 (0.02)	5,98 (0.00)	4,72 (0.08)	5,45 (0.10)	5,38 (0.03)	4,46 (0.00)	4,89 (0.14)	5,17 (0.04)	4,89 (0.00)	5.06	0.48
LA	5,03 (0.43)	5,05 (0.58)	5,17 (0.53)	5,00 (0.54)	5,20 (0.47)	5,05 (0.57)	5,11 (0.61)	5,11 (0.45)	4,94 (0.58)	4,99 (0.)	5.06	0.08
LA-CT gap	0,10	0,53	-0,81	0,28	-0,25	-0,33	0,65	0,22	-0,23	0,10		
REP C2												
Nic*	3,83 (0.13)	3,75 (0.14)	3,60 (0.14)	3,46 (0.17)	3,61 (0.17)	3,69 (0.16)	3,77 (0.16)	3,78 (0.17)	3,83 (0.13)	3,55 (0.15)	3.69	0.13
CT	6,27 (0.00)	6,06 (0.00)	5,83 (0.00)	5,60 (0.00)	5,60 (0.00)	5,06 (0.11)	5,85 (0.00)	5,74 (0.00)	5,67 (0.00)	5,69 (0.00)	5.74	0.32
LA	5,05 (0.59)	5,02 (0.60)	5,28 (0.58)	4,89 (0.60)	4,76 (0.58)	4,99 (0.49)	4,93 (0.59)	4,90 (0.59)	5,03 (0.60)	4,94 (0.60)	4.98	0.14
LA-CT gap	-1,22	-1,04	-0,55	-0,71	-0,84	-0,07	-0,92	-0,84	-0,64	-0,75		

Suppl. Table 5 Vertical Transition Energies and Oscillator Strengths in brackets at XMS-CASPT2, with active-space of |4,4| for 10 solvated representatives of three most populated conformations denoted as REP C0-,C1 and C2 from REMD, along with the average (AVG) and standard deviation (σ) over the 10 representatives.

Suppl. Note. 7 Diabatization of LE and CT states

The LVC Hamiltonian for coupled for a coupled set of diabatic electronic states|d⟩=(|d₁⟩,|d₂⟩, ...,|d_N⟩) may be written as

$$H = \sum_i (K + V_{ii}^{\mathrm{d}}(\boldsymbol{q}) \, |d_i\rangle\langle d_i|) + \sum_{i,j>i} V_{ij}^{\mathrm{d}}(\boldsymbol{q})(|d_i\rangle\langle d_j| + |d_j\rangle\langle d_i|) \qquad (1)$$

where **q** are the dimensionless normal mode coordinates, defined on the ground electronic state S0, with conjugate momenta **p**. The kinetic *K* and potential *V* terms of the Hamiltonian are defined as

$$K = \frac{1}{2}\, \boldsymbol{p}^{\mathrm{T}}\boldsymbol{\Omega}\boldsymbol{p} \quad (2)$$

$$V_{ii}^{\mathrm{d}}(\boldsymbol{q}) = E_{ii}^{\mathrm{d}}(0) + \lambda_{ii}^{\mathrm{T}}(\boldsymbol{q}) + \frac{1}{2}\boldsymbol{q}^{\mathrm{T}}\boldsymbol{\Omega}\boldsymbol{q} \quad (3)$$

$$V_{ij}^{\mathrm{d}}(\boldsymbol{q}) = E_{ij}^{\mathrm{d}}(0) + \lambda_{ij}^{\mathrm{T}}(\boldsymbol{q}) \qquad (4)$$

with **Ω** being the diagonal matrix of normal mode frequencies ω_α , λ_{ij} being the vector of linear coupling constants, $E_{ii}^{\mathrm{d}}(0)$ being the diabatic energy of state *i* at the reference geometry (0 = Franck-Condon), and $E_{ii}^{\mathrm{d}}(0)$ being an electronic coupling constant between diabatic states *i* and *j* at the reference geometry (Franck-Condon). The coupling of electronic states at the reference geometry is the excitonic like term and referred to as **Electronic Coupling** in this work. The λ_{ij} term induces additional coupling between the electronic states due to normal modes **q** and is referred in this work as **Vibronic Coupling**.

The adiabatic states at XMS-CASPT2|4,4| level were diabatized to obtain pure LE (Nic*, La) and CT states. The diabatization was done by diagonalizing the matrix of Fragment Excitation Difference as outlined by Hsu et al.[12] In this method an excitation matrix Δx_{mn} is built, where the matrix elements are difference of excitation density ($\rho^{(mn)}$) between donor and acceptor.

$$\Delta \mathrm{x_{mn}} = \int_{\mathrm{r\epsilon D}} \rho_{\mathrm{ex}}^{(\mathrm{mn})}(\mathrm{r})\mathrm{dx} - \int_{\mathrm{r\epsilon A}} \rho_{\mathrm{ex}}^{(\mathrm{mn})}(\mathrm{r})\mathrm{dx} \qquad (5)$$

For the diagonal elements the excitation density $\rho^{(mm)}$ is defined as sum of attachment and detachment densities, while for off-diagonal elements $\rho^{(mn)}$ is defined as the transition density between the states.

To conduct this protocol, the 1-electron transition density matrix (TDM) in molecular-orbital (MO) basis was obtained using the RASSI module of Openmolcas. To obtain attachment/detachment/transition densities localized on the fragment monomers, the TDM was converted from MO to atomic-orbital (AO) basis and the coefficients in this AO basis were summed to obtain the fraction of densities on two monomers.

The non-diagonal symmetric Δx_{mn} matrix obtained was diagonalized to give the rotation matrix **D** needed to transform the adiabatic states at Franck-Condon geometry [$\boldsymbol{q}$ = 0] to diabatic states.

$$\Delta \mathrm{x}_{\mathrm{mn}}^{diag} = \mathrm{D}^{-1}\Delta \mathrm{x_{mn}}\mathrm{D} \quad (6)$$

This matrix **D** can be applied to transform the diagonal Hamiltonian in the adiabatic basis ψ at [$\boldsymbol{q}$ = 0] to a non-diagonal symmetric Hamiltonian matrix in diabatic basis ϕ at [$\boldsymbol{q}$ = 0] with energies and

interstate electronic couplings of diabatic LE and CT states. We utilized a *crude-diabatic* (in reference to crude-adiabatic approximation[13]) approximation for our quantum dynamics where these frozen diabatic states at Franck-Condon geometry ϕ[**q** = 0] are the reference states amongst which the population transfer is monitored.

$$\mathrm{H}_{\phi[\boldsymbol{q}=0]} = \mathrm{D}^{-1}\mathrm{H}_{\psi[\boldsymbol{q}=0]}\mathrm{D} \qquad (7)$$

The above procedure gives us the energies of diabatic states and 0th-order coupling of the LVC Hamiltonian, the coupling between diabatic states induced due to relative orientation the bases at FC geometry. This coupling is also referred as **Electronic Coupling** in this work.

To obtain the linear coupling constants λ_{ij}, we displace each normal coordinate α by some small value $\pm\Delta_\alpha$ and compute the energies of the adiabatic states giving us a diagonal Hamiltonian at **q** = Δ_α. Then using two transformations as described below, this diagonal Hamiltonian is transformed to non-diagonal symmetric Hamiltonian in the previously described diabatic basis ϕ[**q** = 0].

The overlap matrix **S** between the adiabatic states at displaced geometry along a normal mode [***q*** = Δ_α] and adiabatic states at Franck-Condon [***q*** = 0] was computed. This overlap matrix **S** was used to transform the diagonal Hamiltonian at displaced geometry to non-diagonal symmetric Hamiltonian in the basis of adiabatic states at [***q*** = 0]. Following this, this non-diagonal symmetric Hamiltonian in adiabatic basis of [***q*** = 0] was then further transformed to Hamiltonian in the basis of diabatic states at [***q*** = 0] through the previously computed rotation matrix **D.**

$$\begin{aligned}\mathrm{H}_{\phi[\mathrm{Q}_0]} &= \langle\phi[Q_0]|\psi[Q_0]\rangle\,\langle\boldsymbol{\psi}[\boldsymbol{Q_0}]|\boldsymbol{\psi}[\boldsymbol{Q}]\rangle\,\mathrm{H}_{\psi[\mathrm{Q}=\Delta_\alpha]}\,\langle\boldsymbol{\psi}[\boldsymbol{Q_0}]|\boldsymbol{\psi}[\boldsymbol{Q}]\rangle^{-\mathbf{1}}\,\langle\phi[Q_0]|\psi[Q_0]\rangle^{-1} \\ &= \mathrm{D}\,\mathbf{S}\,\mathrm{H}_{\psi[\mathrm{Q}]}\,\mathbf{S}^{-\mathbf{1}}\,\mathrm{D}^{-1} \qquad (8)\end{aligned}$$

The above procedure gives the gradients and additional couplings of the diabatic states induced by motion along the normal mode [Q_j] , also known as **Vibronic coupling.**

7.1 Electronic Coupling (V), Energy-gap (ΔE) and Mixing (V / ΔE) values for 100 different solute conformations of most populated cluster C0.

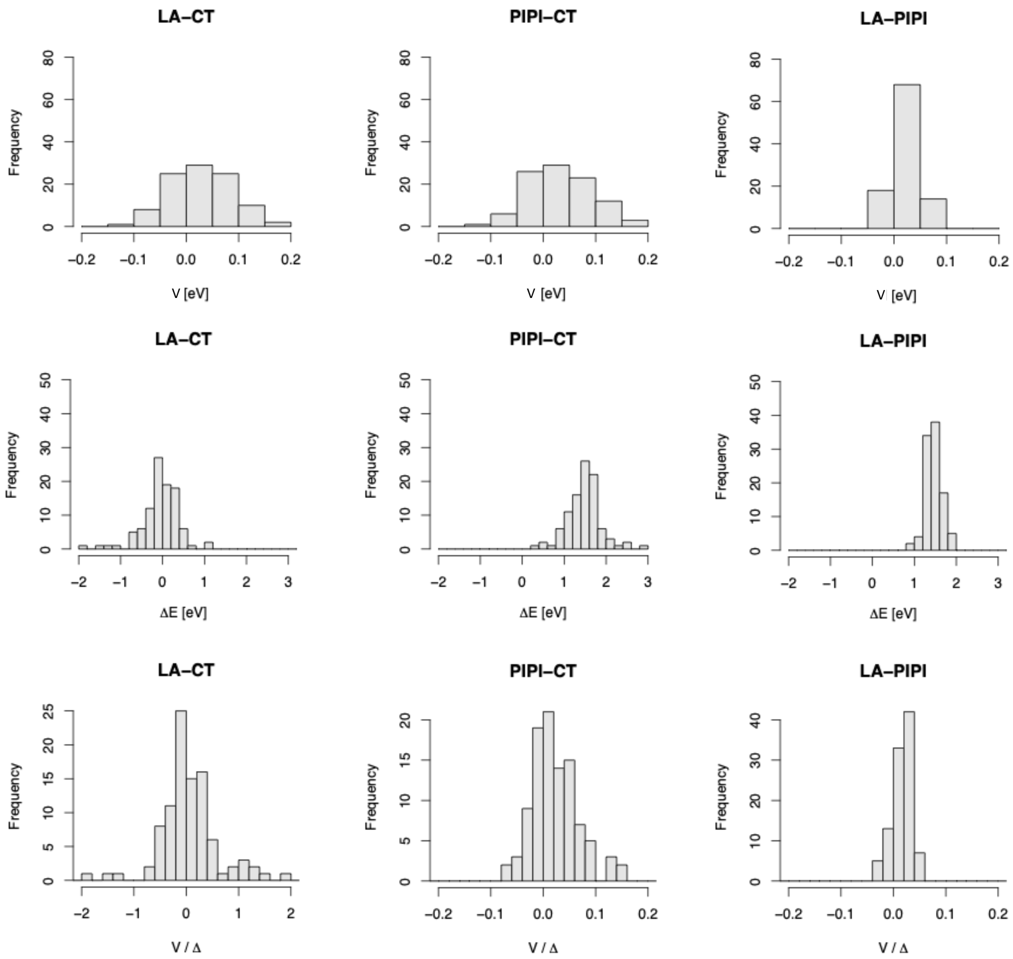

Suppl. Fig. 8 Electronic coupling, Energy gap , and mixing (V/delta) for 100 different solute conformations of most representative cluster in solvent. These are obtained by diabatizing the adiabatic states computed at |4,4|/XMS-CASPT2 level.

7.2 Adiabatic and diabatic states of frozen solute in 100 solvent ensemble.

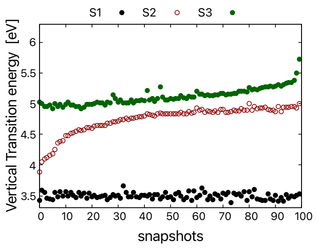

ADIABATIC

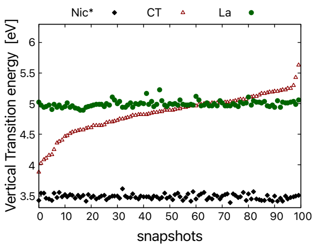

DIABATIC

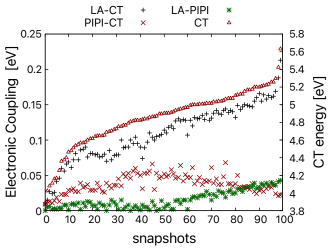

COUPLING

Suppl. Fig. 9 Adiabatic, Diabatic states and Electronic coupling of a fixed solute geometry from most populated cluster in 100 different equilibrated solvent ensembles

Suppl. Note. 8 Wavepacket dynamics on Cluster representatives

ù

8.1 LVC parameters for dynamics on Cluster representatives in diabatic basis

	**Nic*** (eV)	**CT** (eV)	**La** (eV)	**La-CT** (eV)	**CT-Nic*** (eV)	**La-Nic*** (eV)
**Cluster C0**						
1	3.82	4.63	5.00	-0.06	0.01	0.01
2	3.57	5.12	5.01	0.01	0.06	-0.04
3	3.74	5.43	4.95	0.00	0.00	0.00
4	3.60	4.74	4.97	0.00	0.00	-0.01
5	3.74	4.77	5.01	0.06	0.02	-0.04
6	3.43	3.81	4.87	0.01	-0.02	-0.14
**Cluster C1**						
1	3.71	5.97	5.17	0.00	0.06	0.03
2	3.92	4.75	4.96	-0.02	-0.09	-0.03
3	3.67	5.39	5.25	0.02	0.03	0.10
4	3.98	4.39	5.01	0.00	0.00	0.00
5	3.90	5.14	4.95	-0.05	0.06	0.03
6	4.10	4.88	4.98	-0.02	0.00	0.03
**Cluster C2**						
1	3.75	6.05	5.01	0.00	0-.01	0.00
2	3.45	5.59	4.88	0.00	0.00	0.00
3	3.61	5.59	4.75	-0.01	0.00	0.00
4	3.69	5.06	4.99	0.00	0.00	0.00
5	3.77	5.73	4.89	0.01	-0.02	0.03
6	3.54	5.68	4.94	0.00	0.00	0.00

Suppl. Table 6: LVC parameters (vertical excitation energy in columns 2-4 and interstate electronic coupling matrix elements in columns 5-7) for ML-MCTDH dynamics on cluster representatives shown in next section.

8.2 Wavepacket dynamics for CT-mediated EET including all coupling terms in vibronic Hamiltonian in diabatic-basis.

CLUSTER C0

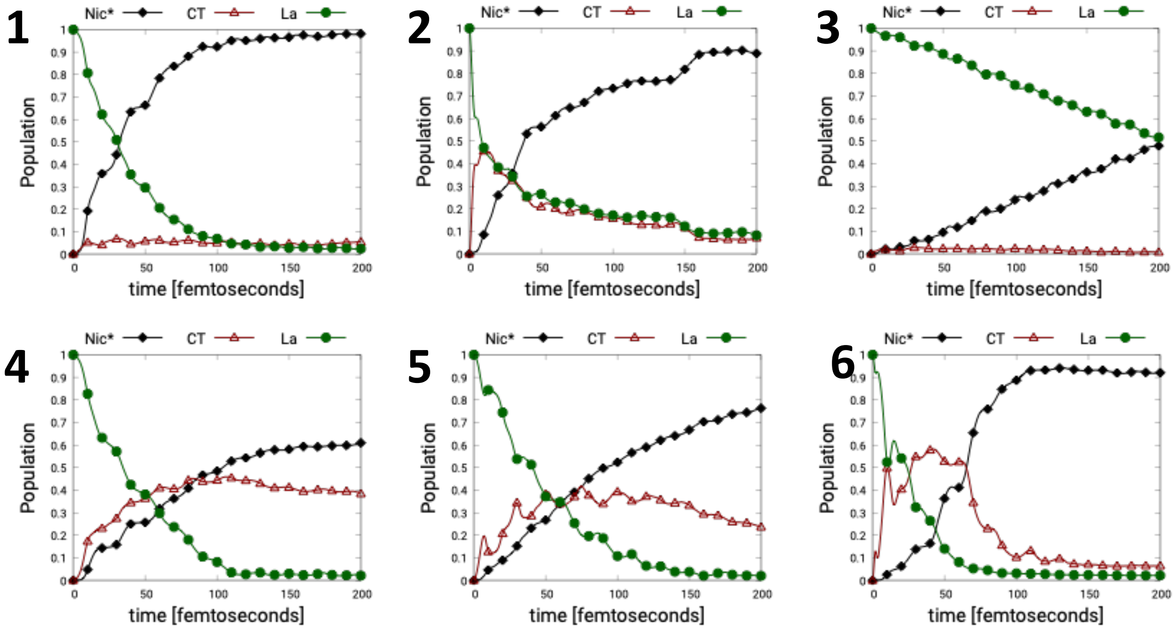

CLUSTER C1

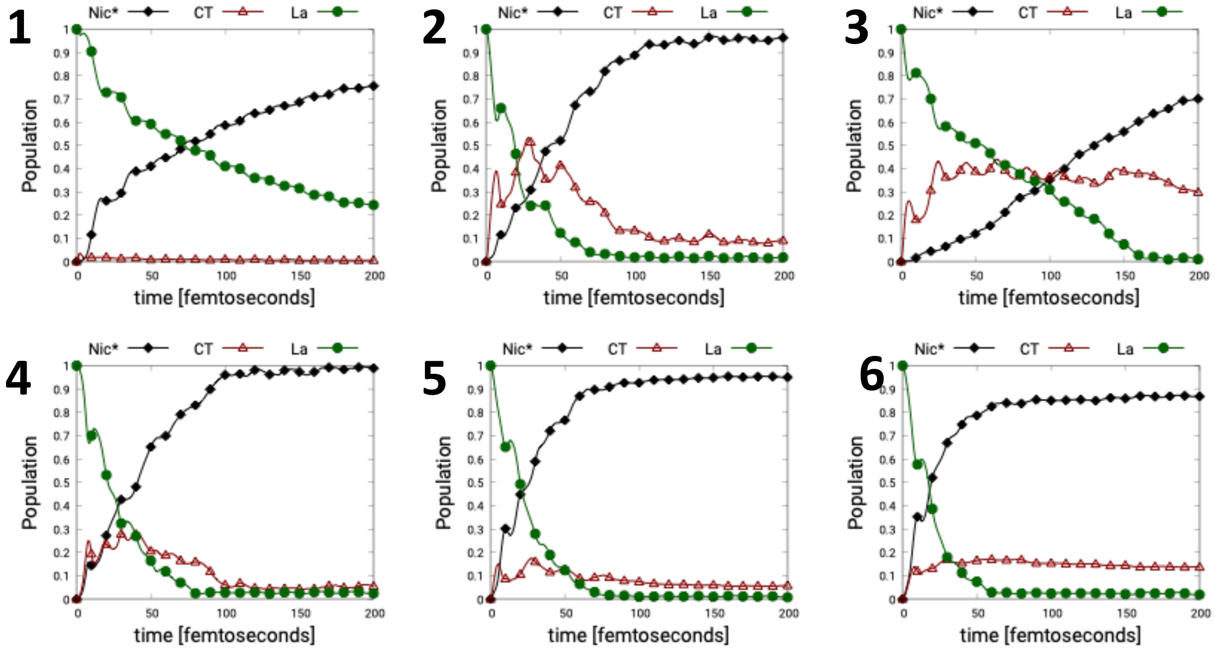

CLUSTER C2

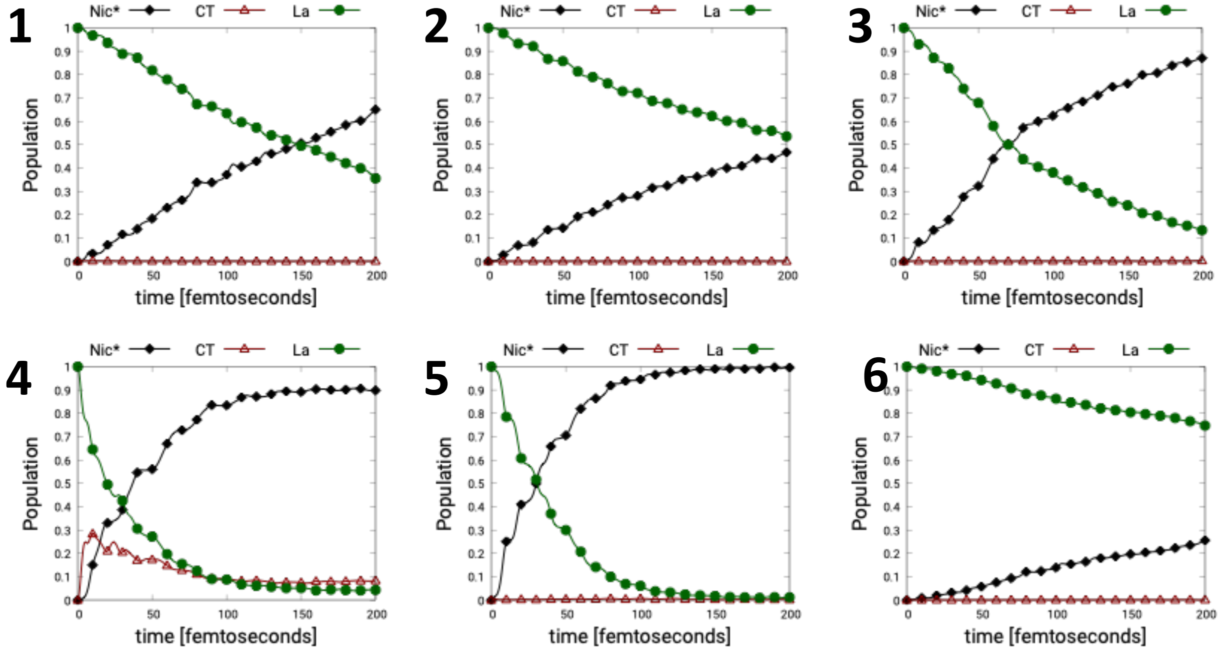

Suppl. Fig. 10: ML-MCTDH dynamics including all 3 electronic states and all coupling and gradient terms. The dynamics are labeled (**1,2** etc) according to Suppl. Table 6

8.3 Wavepacket dynamics for Direct EET including all coupling terms in vibronic Hamiltonian in diabatic-basis.

CLUSTER C0

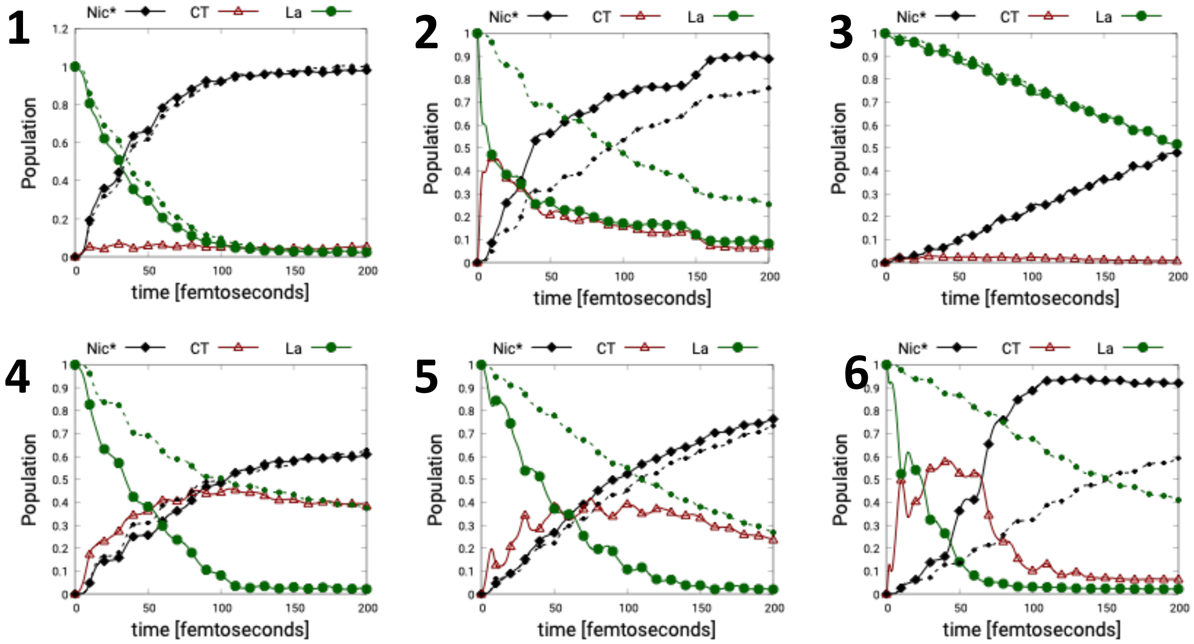

CLUSTER C1

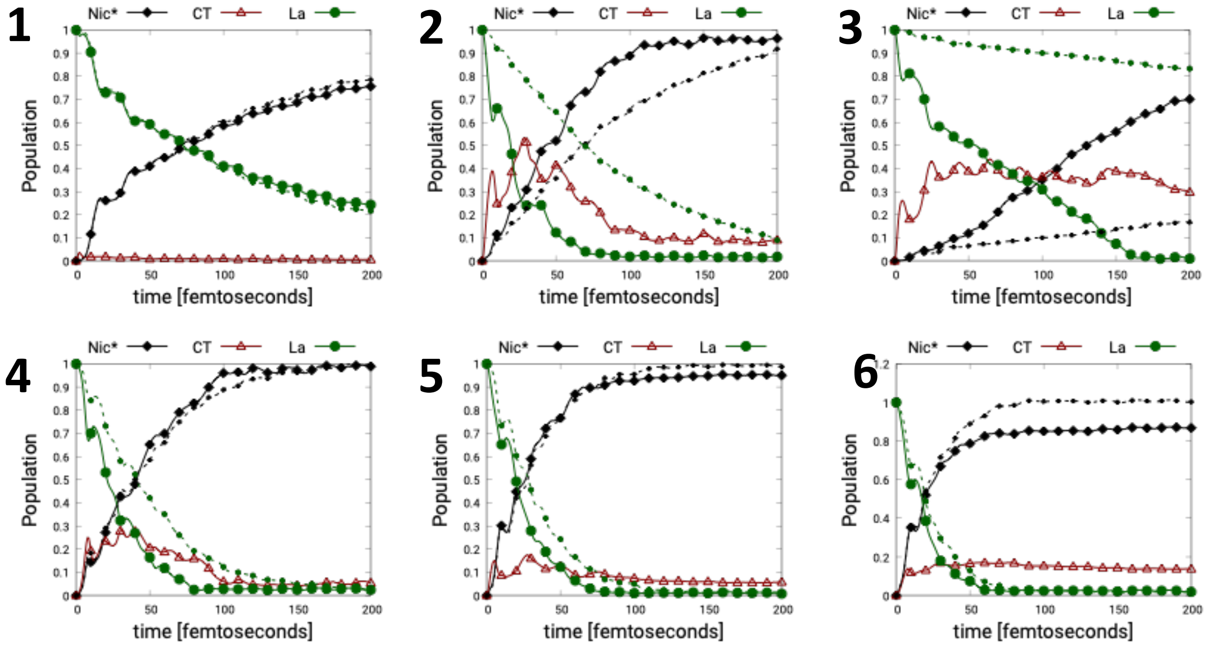

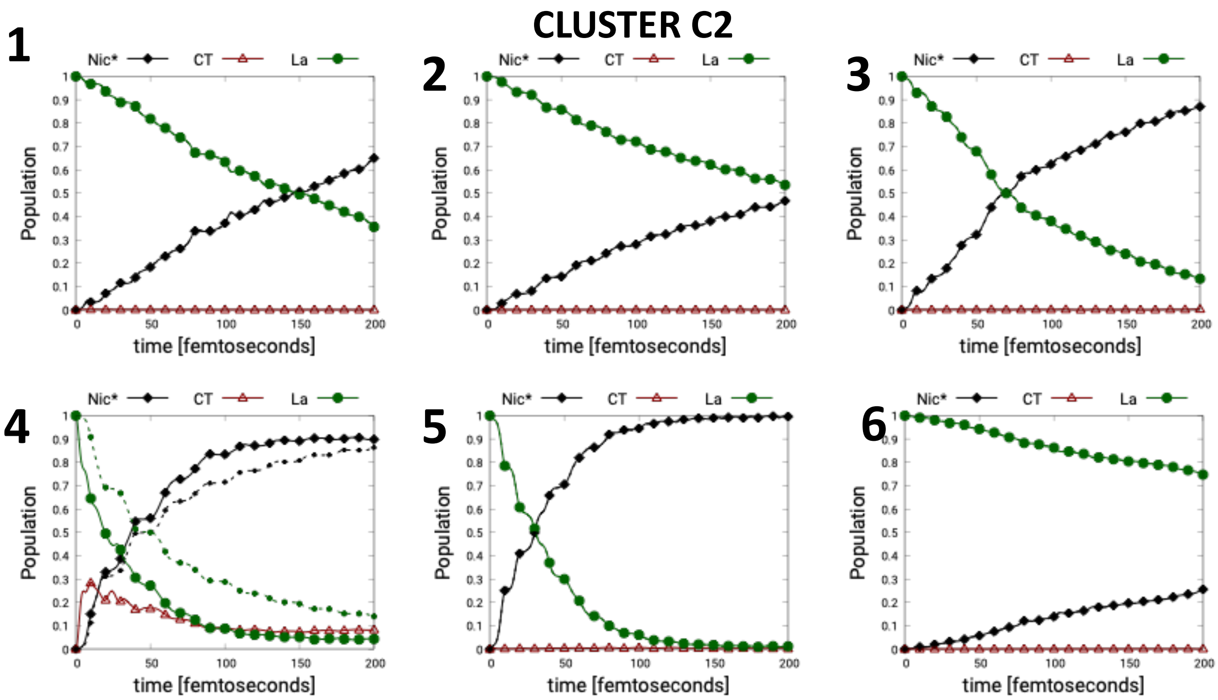

Suppl. Fig. 11: Solid line: ML-MCTDH dynamics with three electronic states and all coupling/gradient terms. Dashed line: ML-MCTDH dynamics with two electronic states (NO CT) simulating Direct Transfer and respective gradient and all interstate coupling terms.

Suppl. Note. 9 Energies of diabatic states along example ML-MCTDH dynamics.1.

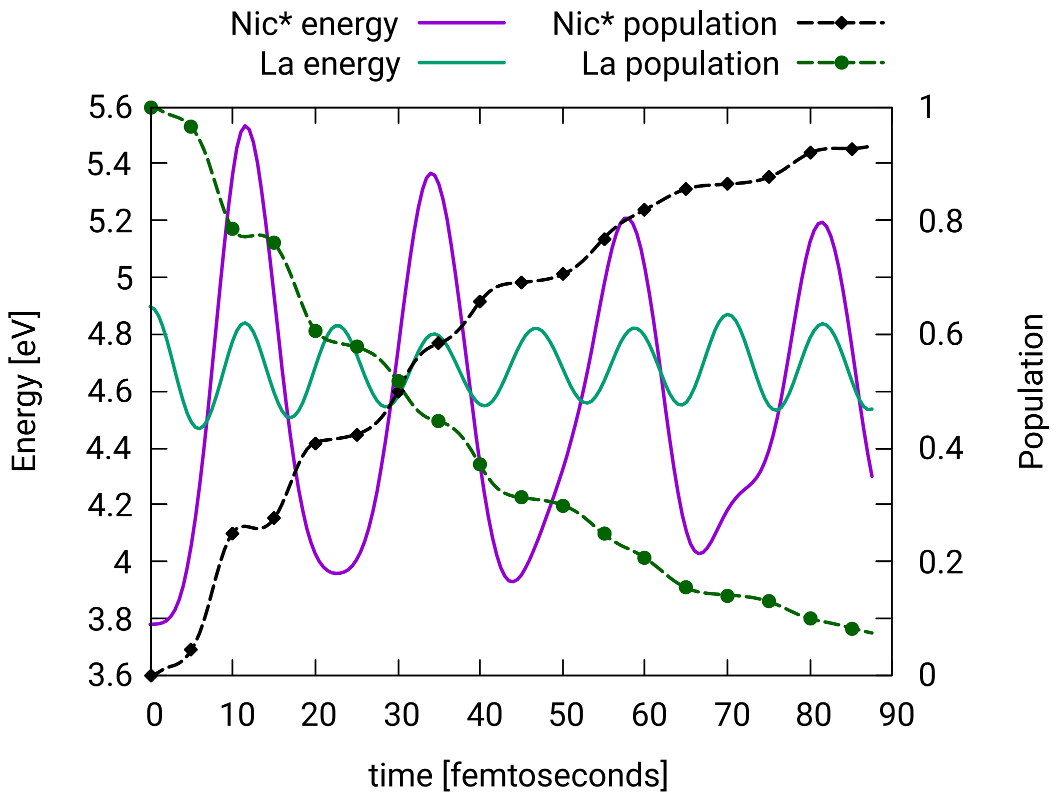

Suppl. Fig. 12: Energies of Nic* and La state along ML-MCTDH dynamics for a representative structure (Cluster C2 / Struct5). The population transfer mainly happens around the times when La and Nic* energies become degenerate.

Suppl. Note. 10 Selective effects of Interstate Electronic/Vibronic Coupling on wavepacket dynamics.

To explore the selective effects of Electronic Coupling (*coupling between diabatic LE/CT states due to inter-base orientation at Franck-Condon geometry*) or Vibronic Coupling (*additional coupling induced due to photo-excited normal modes of the system*), these terms were switched ON or OFF in the Hamiltonian. This is accomplished by removing the respective terms from the operator file in ML-MCTDH dynamics.

Cluster C0
Structure 01

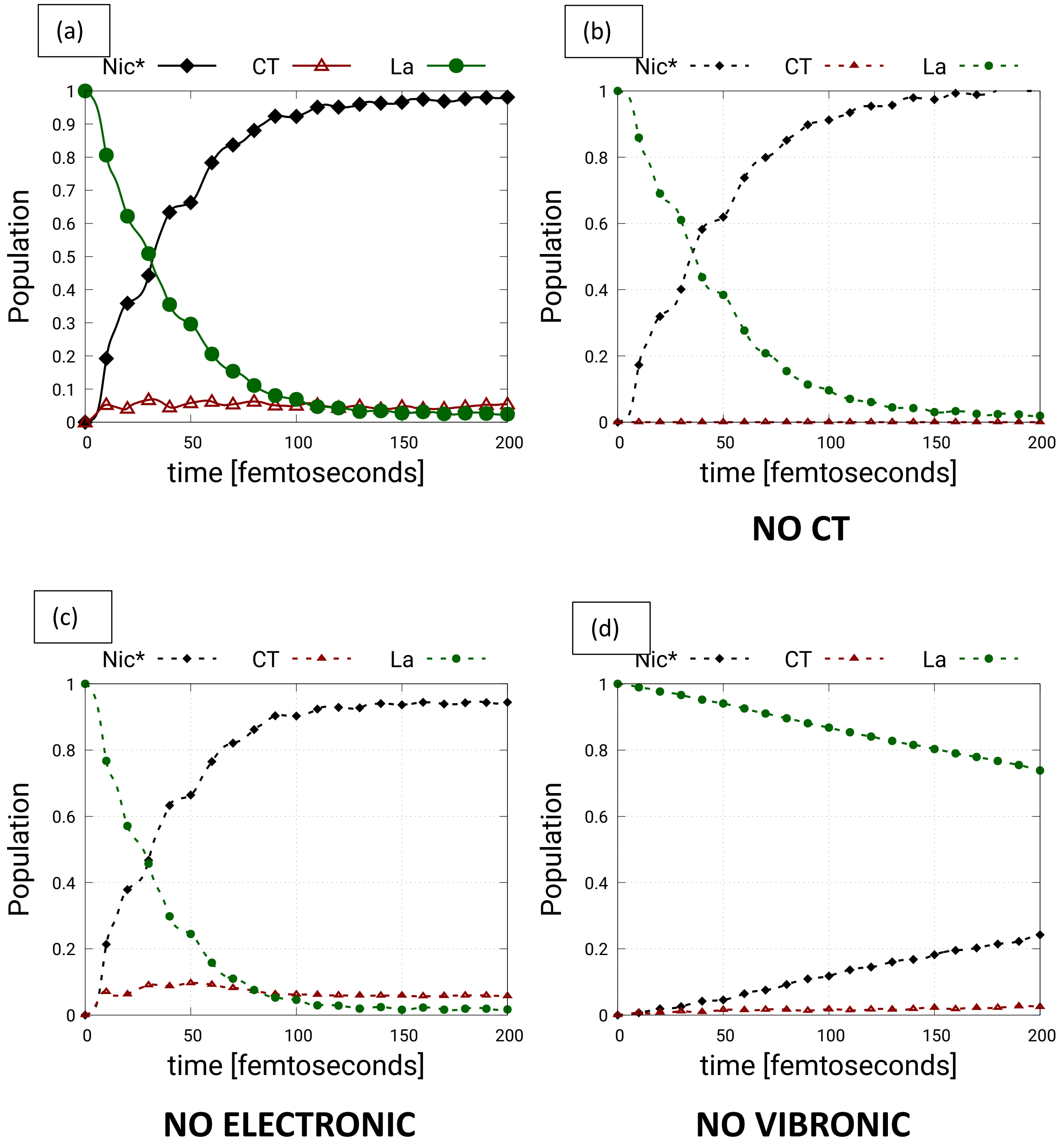

Suppl. Fig. 13.1: (a) ML-MCTDH dynamics will coupling terms and the three electronic states. (b) ML-MCTDH dynamics with all coupling (between L_a and Nic*) terms but NO CT state (c) ML-MCTDH dynamics after removing all electronic coupling terms (off-diagonal terms of electronic hamiltonian) (d) ML-MCTDH dynamics after removing all vibronic terms (gradients and coupling terms of vibronic hamiltonian)

Cluster C0
Structure 02

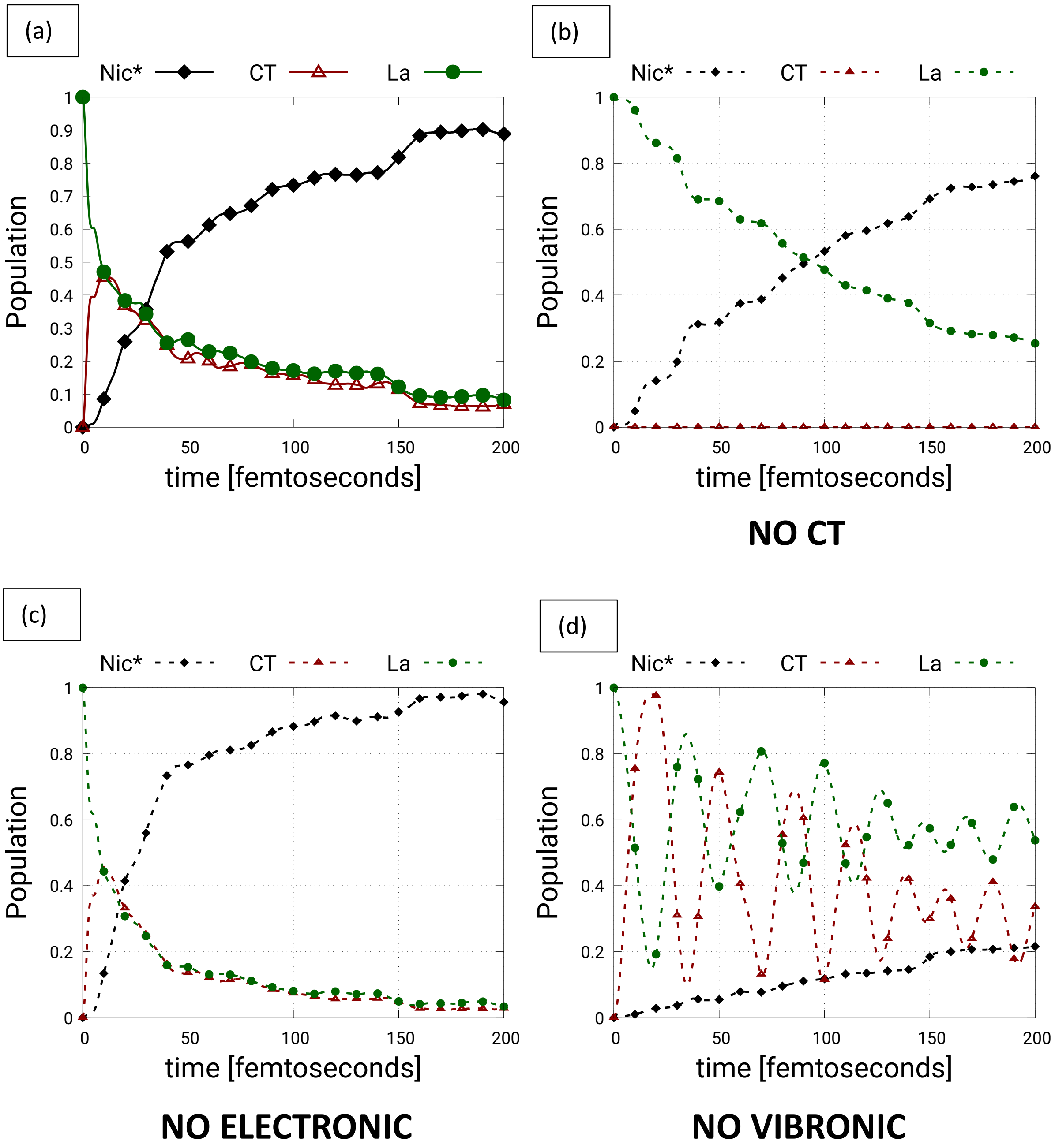

Suppl. Fig. 13.2: Caption same as Suppl. Fig. 13.1

Cluster C0
Structure 03

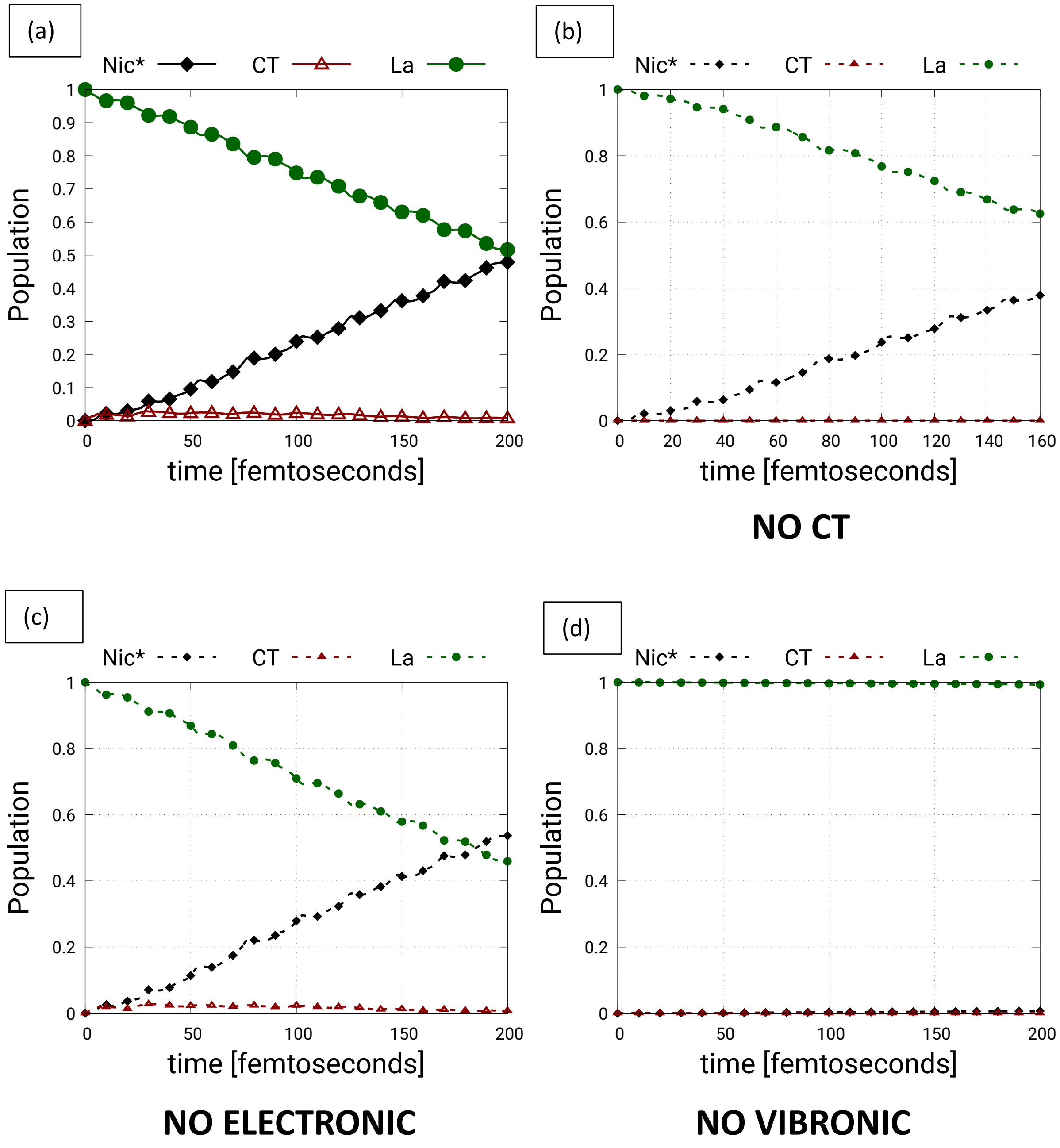

Suppl. Fig. 13.3 : Caption same as Suppl. Fig. 13.1

Cluster C0
Structure 04

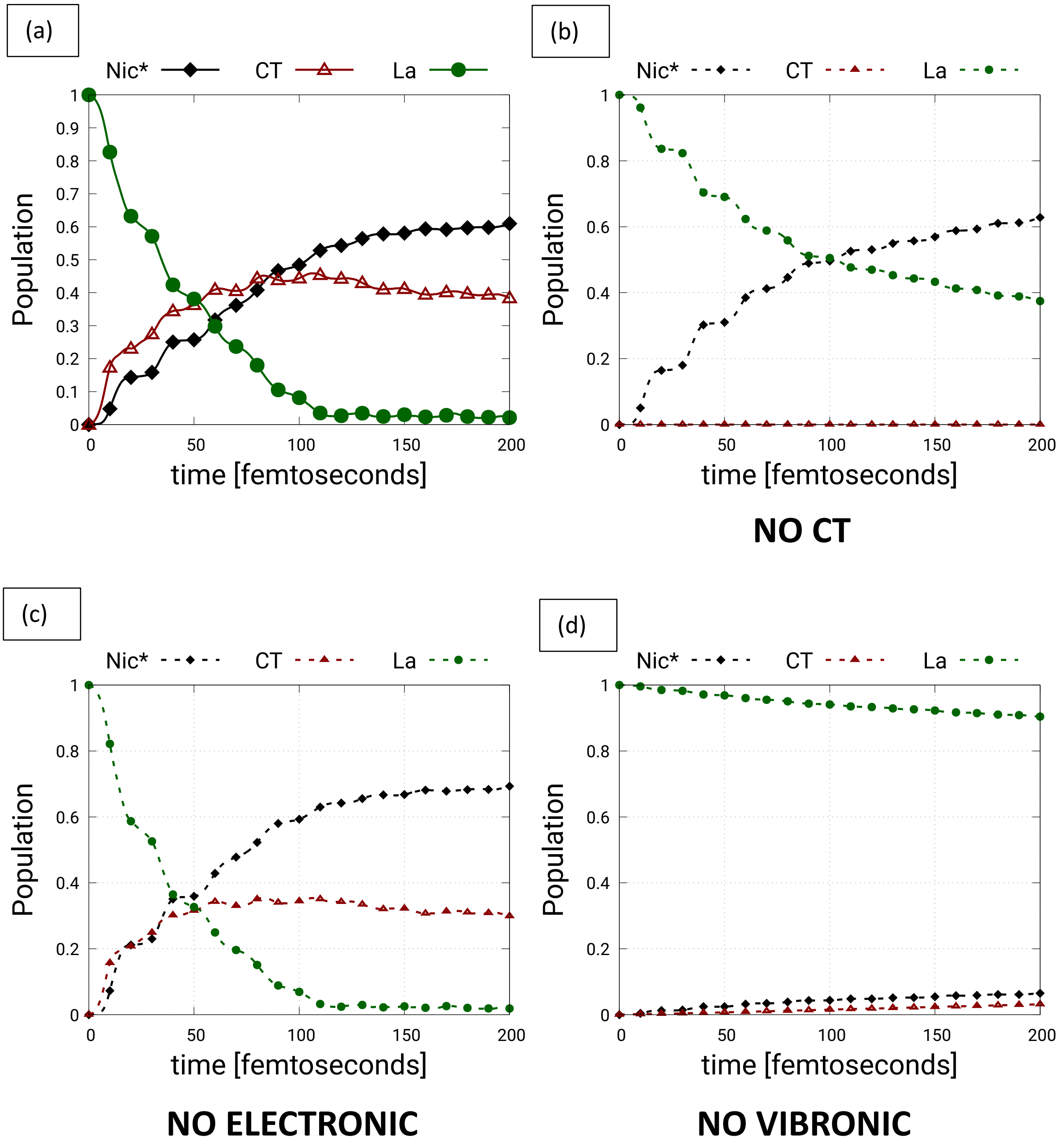

Suppl. Fig. 13.4: Caption same as Suppl. Fig. 13.1

Cluster C0
Structure 05

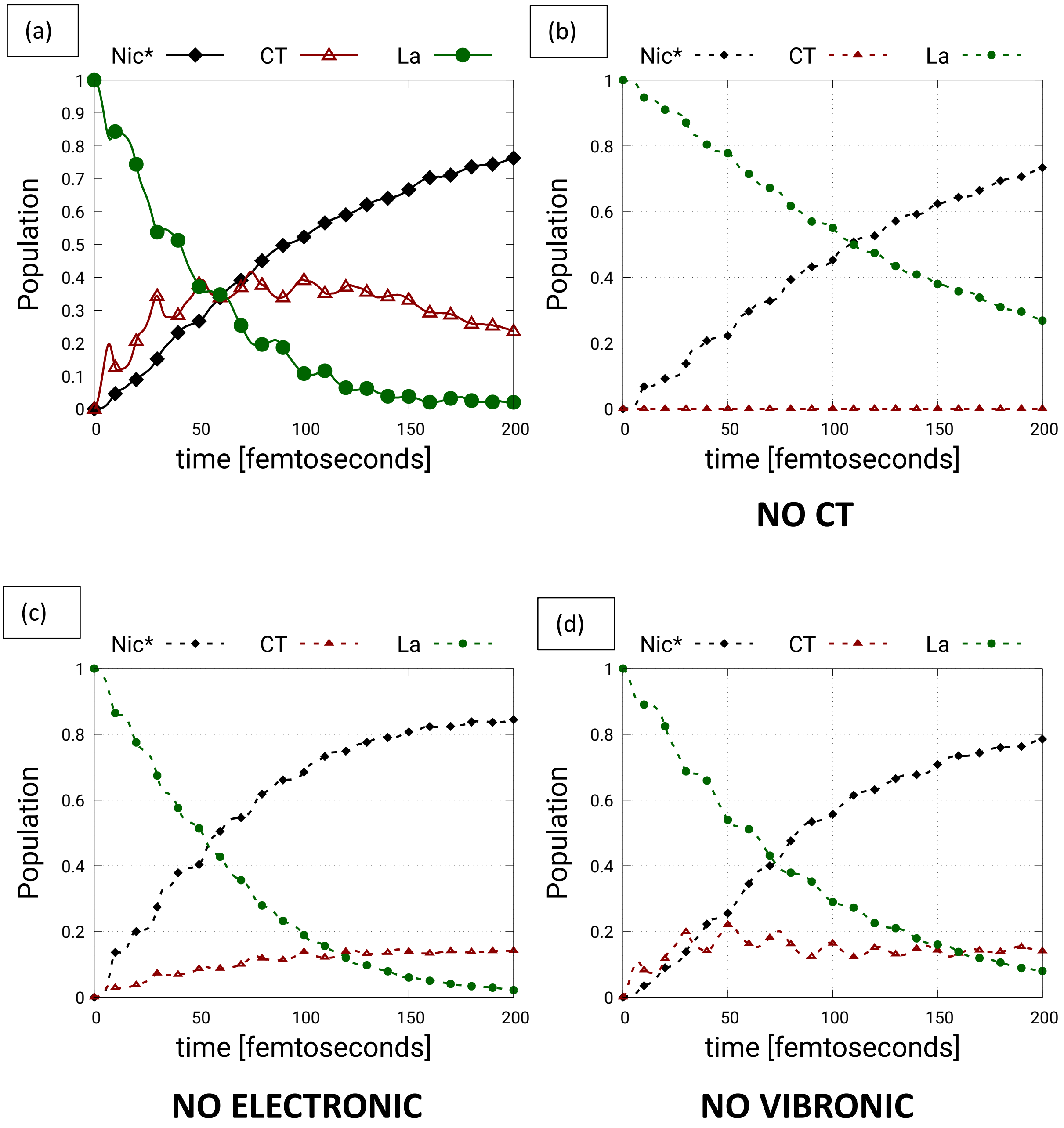

Suppl. Fig. 13.5: Caption same as Suppl. Fig. 13.1

Cluster C0
Structure 06

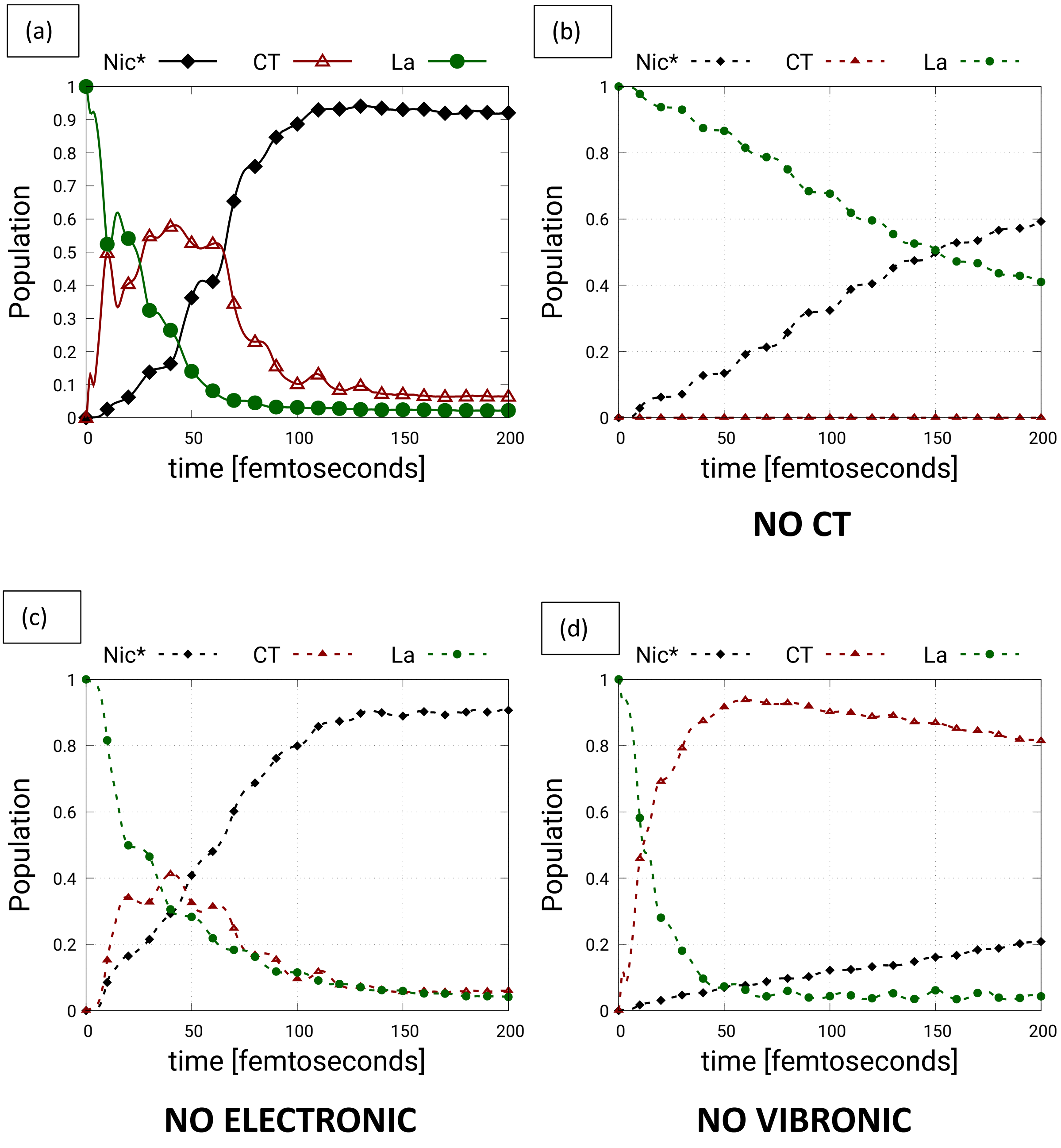

Suppl. Fig. 13.6: Caption same as Suppl. Fig. 13.1

Cluster C1
Structure 01

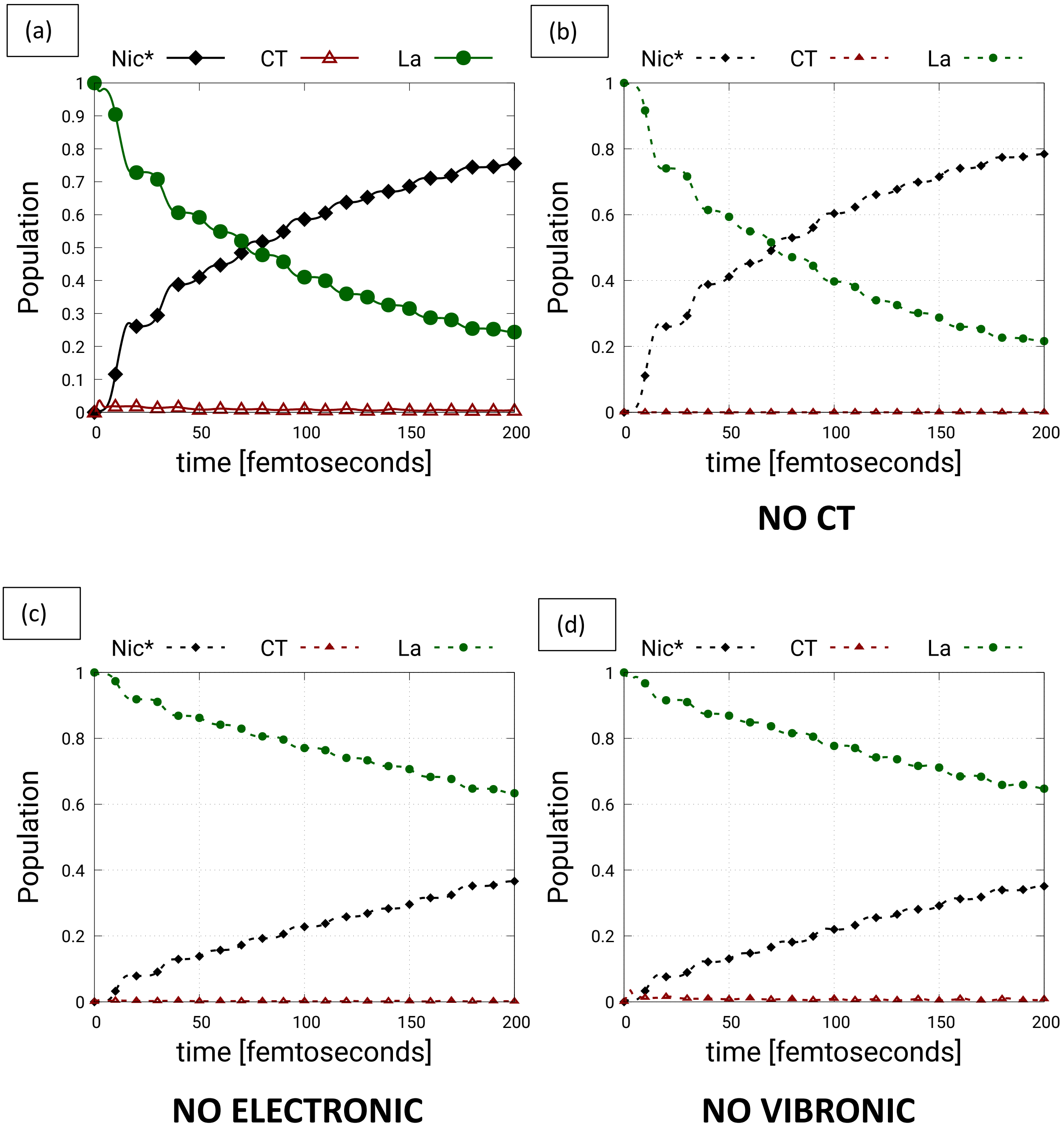

Suppl. Fig. 13.7: Caption same as Suppl. Fig. 13.1

Cluster C1
Structure 02

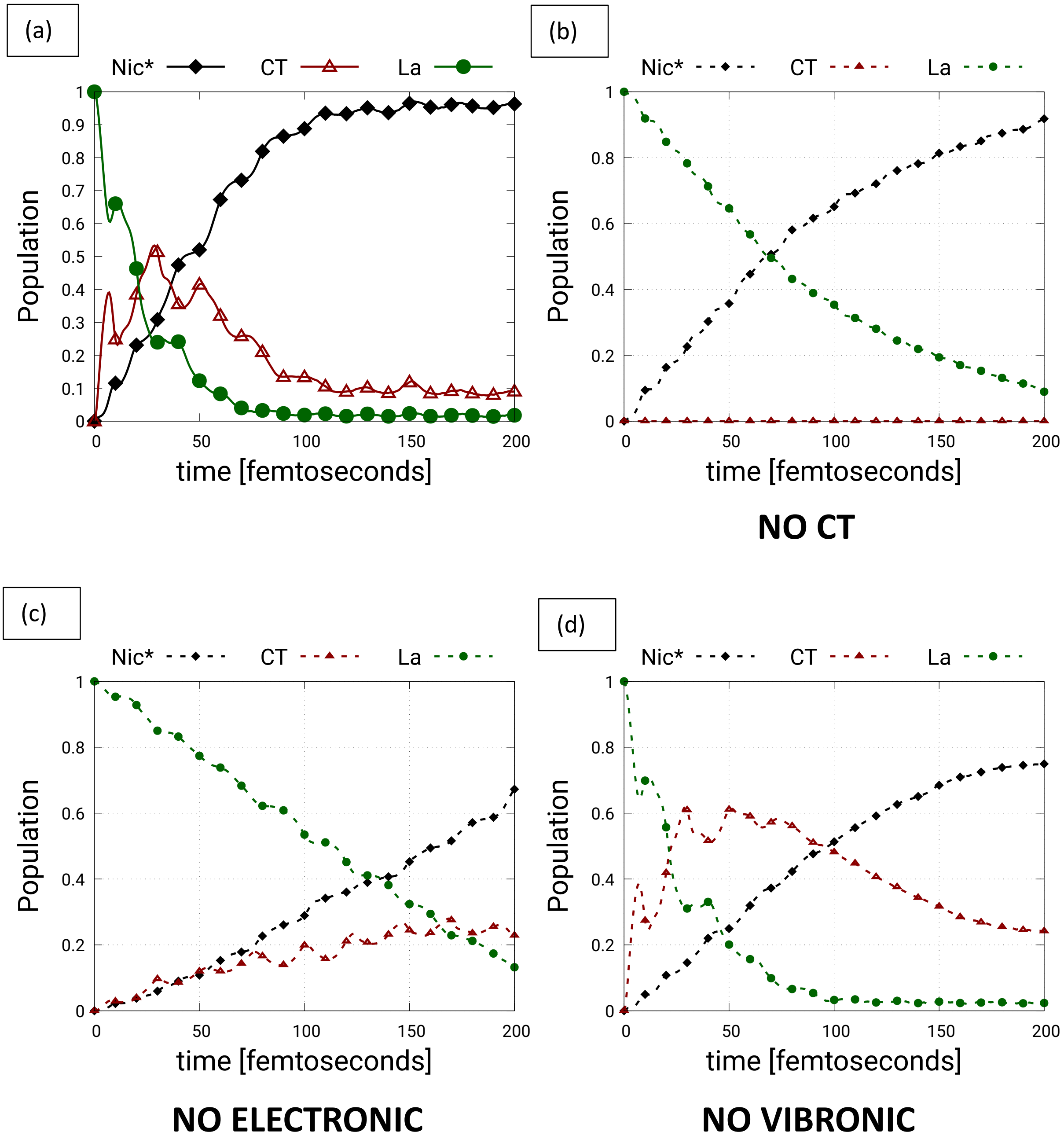

Suppl. Fig. 13.8: Caption same as Suppl. Fig. 13.1

Cluster C1
Structure 03

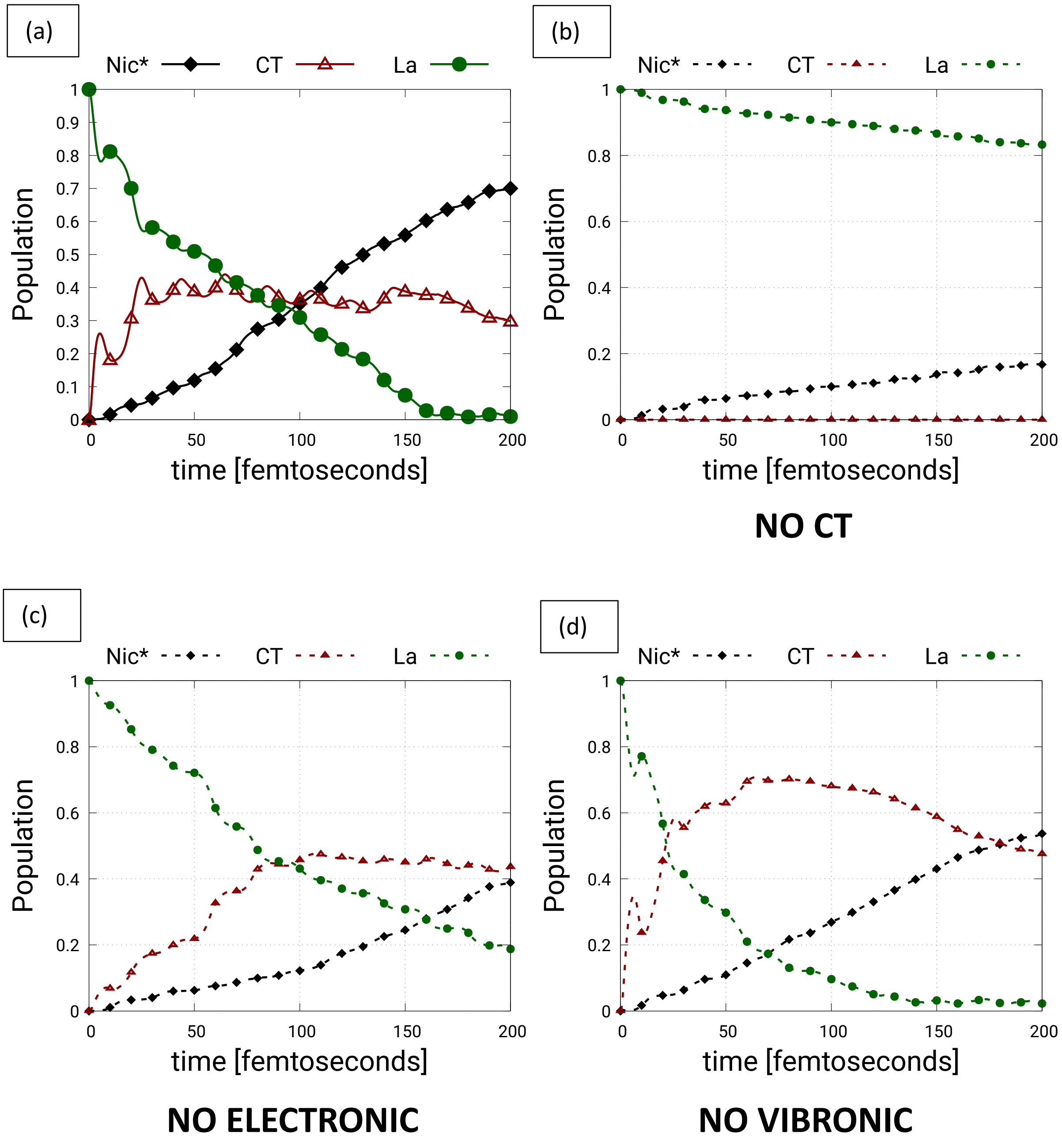

Suppl. Fig. 13.9: Caption same as Suppl. Fig. 13.1

Cluster C1
Structure 04

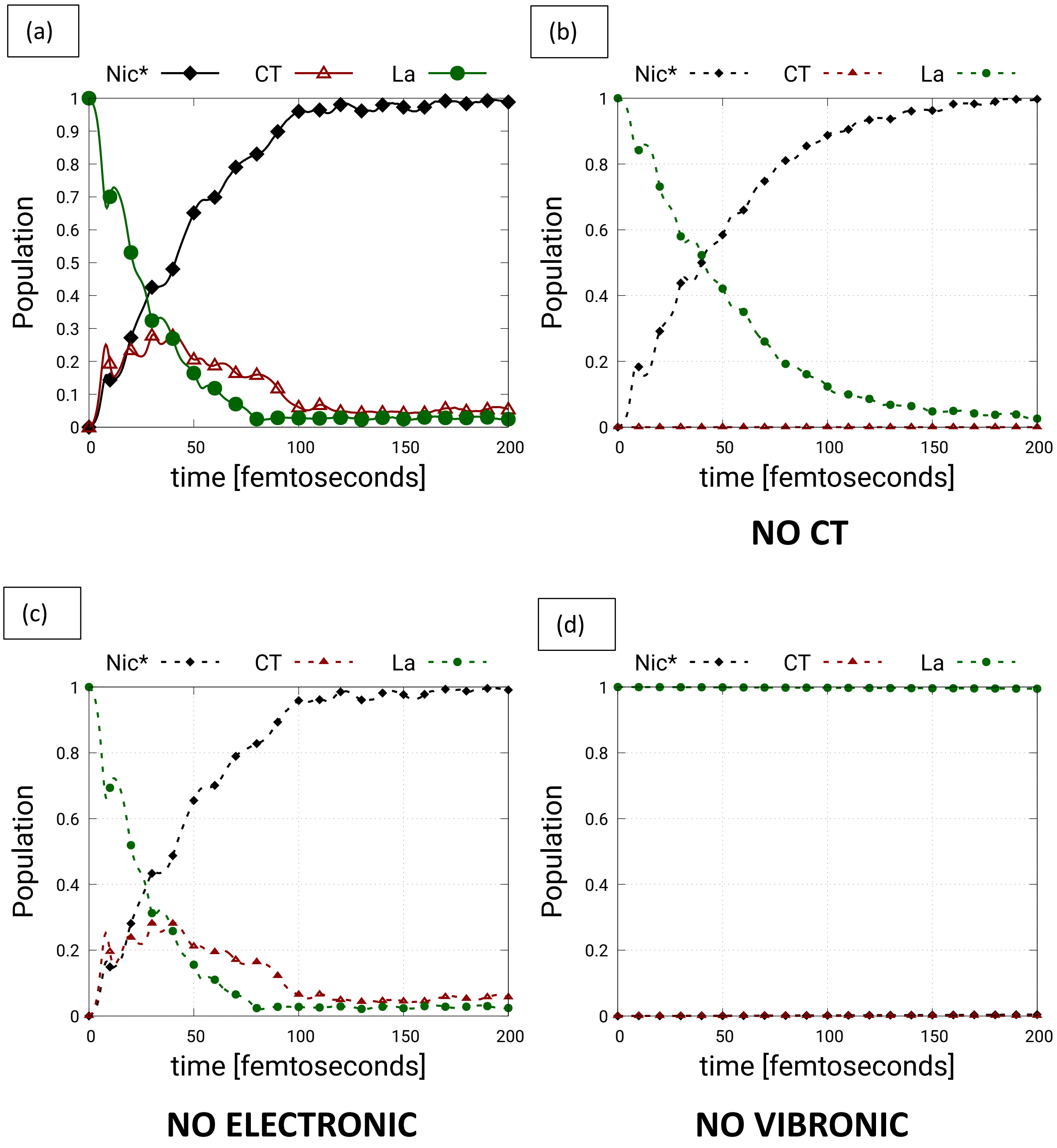

Suppl. Fig. 13.10: Caption same as Suppl. Fig. 13.1

Cluster C1
Structure 05

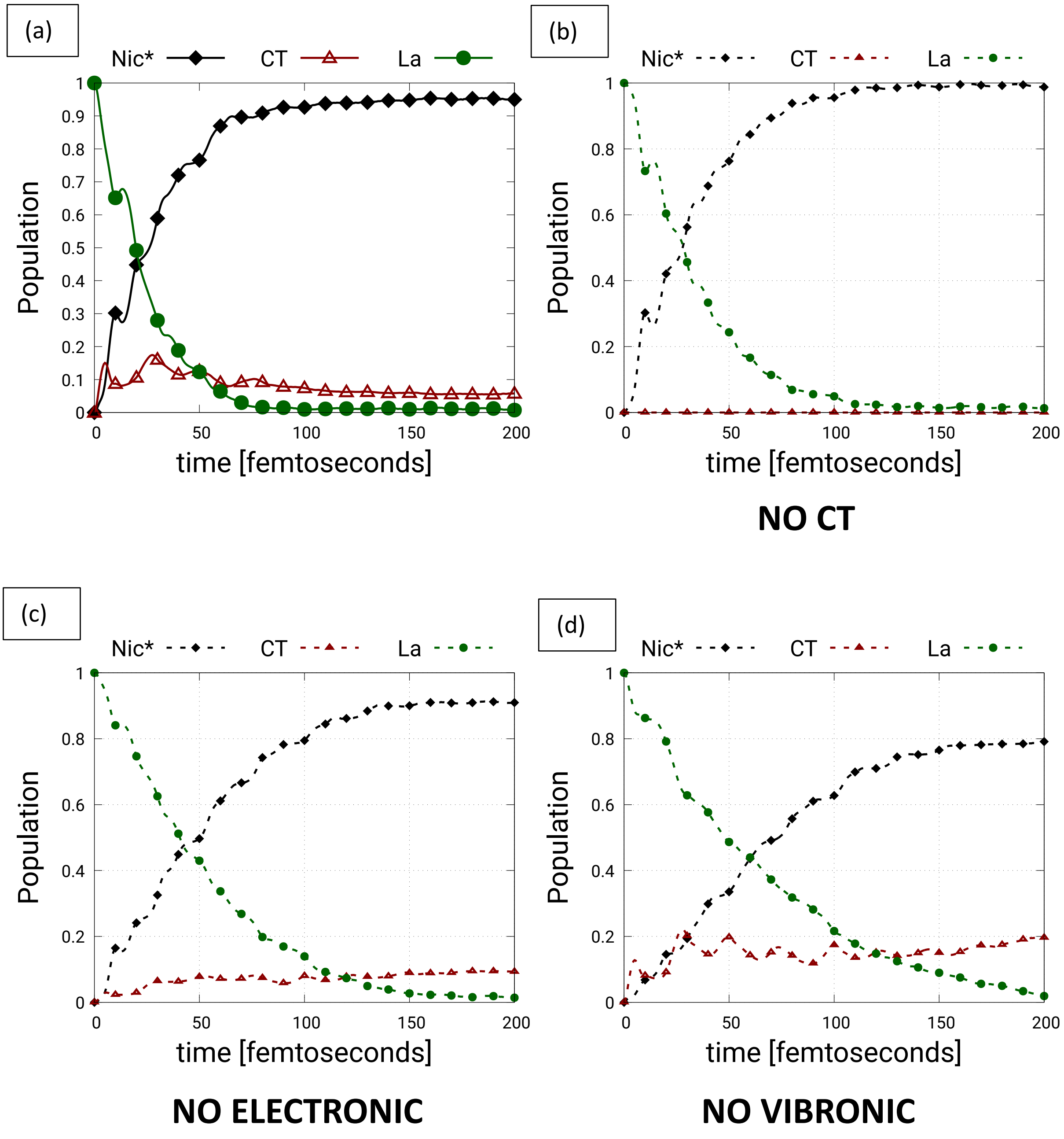

Suppl. Fig. 13.11: Caption same as Suppl. Fig. 13.1

Cluster C1
Structure 06

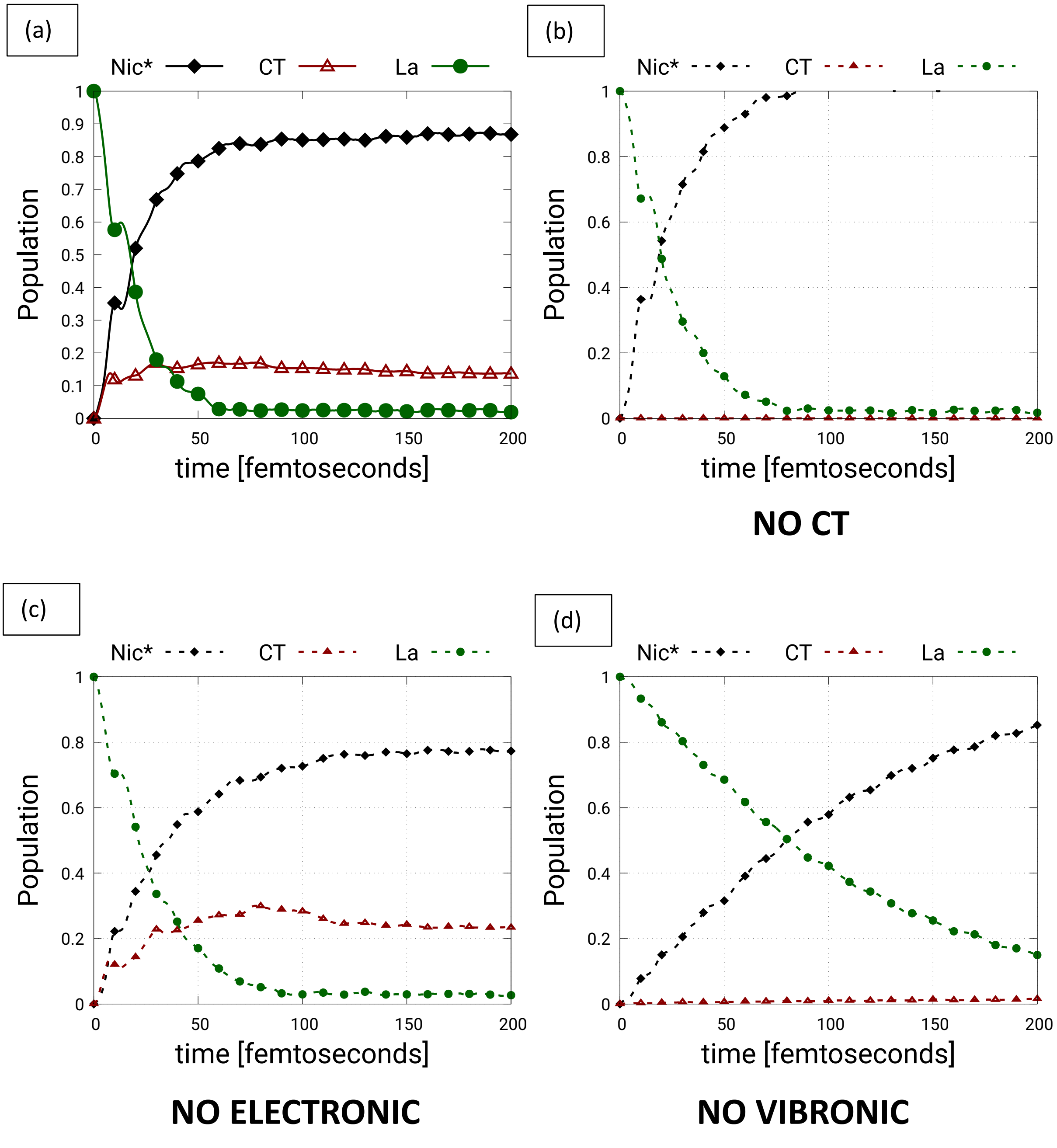

Suppl. Fig. 13.12: Caption same as Suppl. Fig. 13.1

Cluster C2
Structure 01

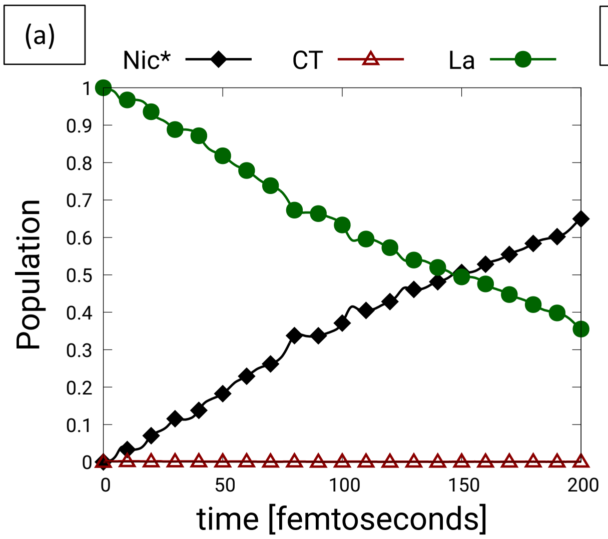

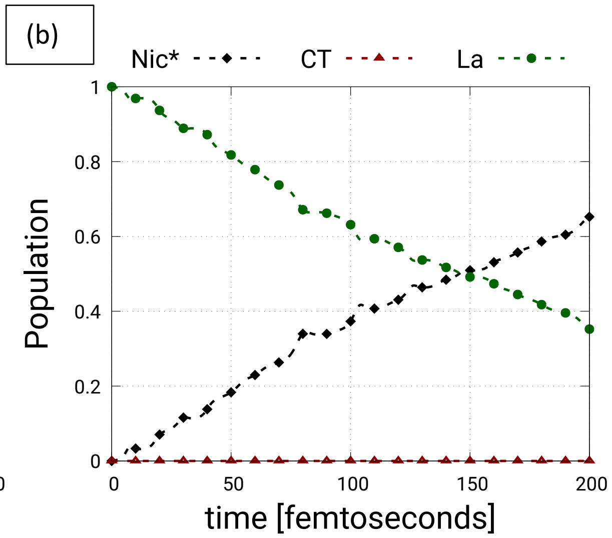

NO CT

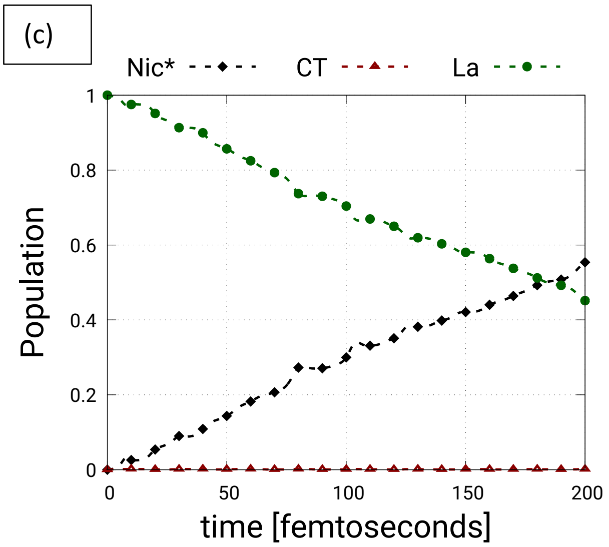

NO ELECTRONIC

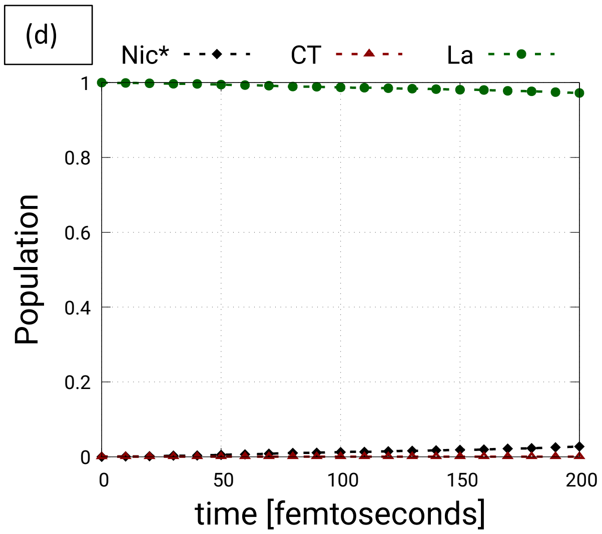

NO VIBRONIC

Suppl. Fig. 13.13: Caption same as Suppl. Fig. 13.1

Cluster C2
Structure 02

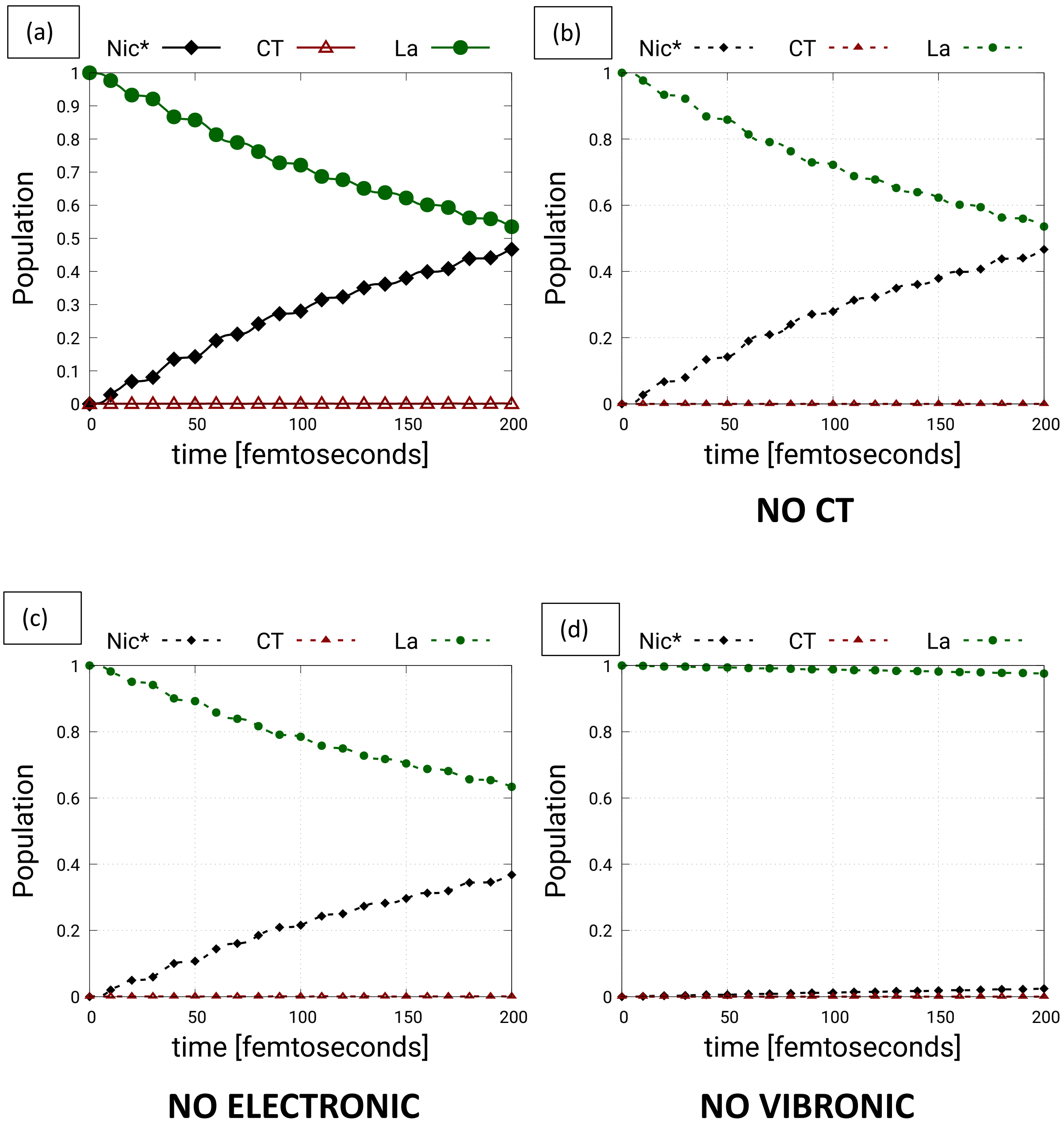

Suppl. Fig. 13.14: Caption same as Suppl. Fig. 13.1

Cluster C2
Structure 03

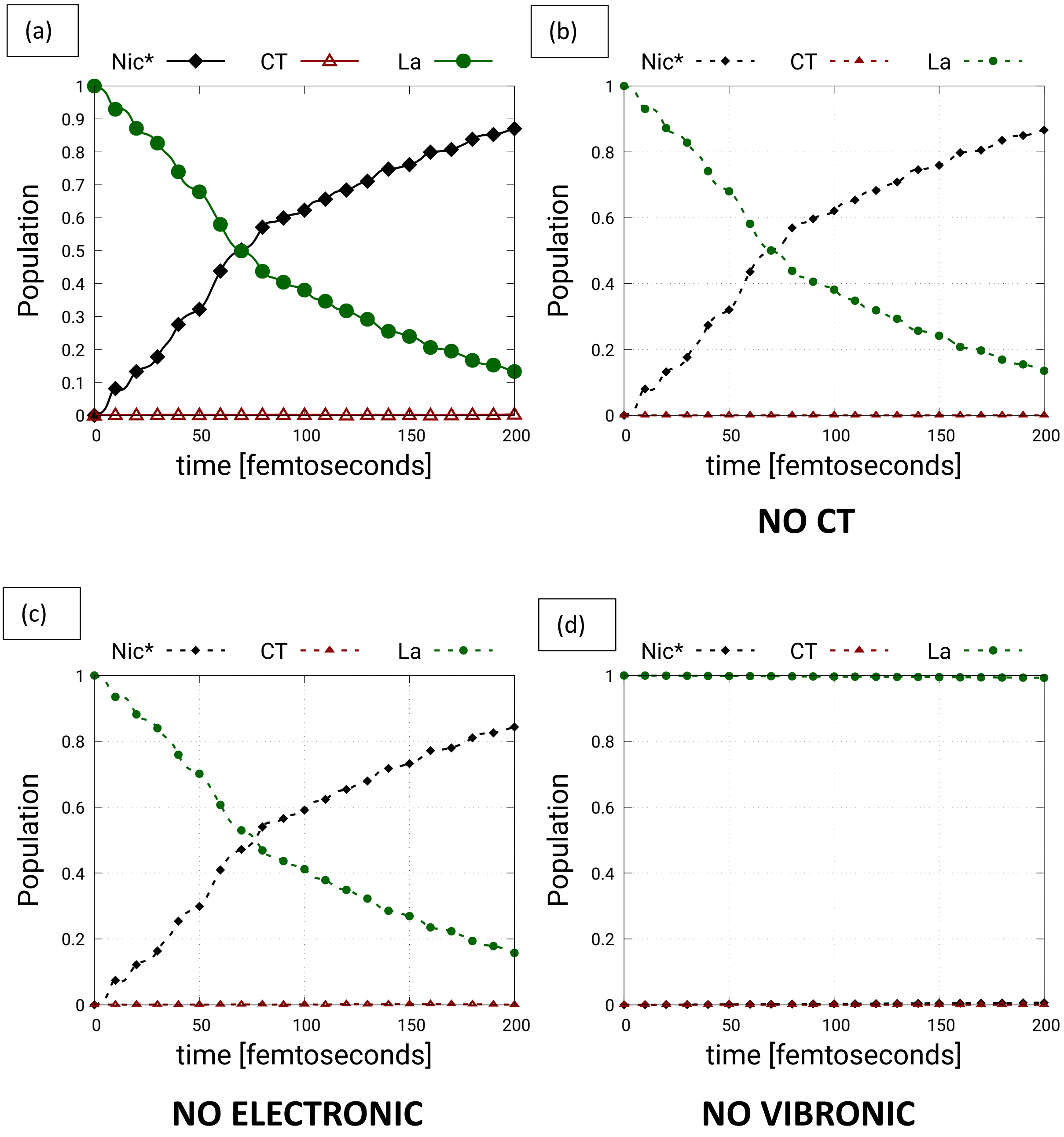

Suppl. Fig. 13.15: Caption same as Suppl. Fig. 13.1

Cluster C2
Structure 04

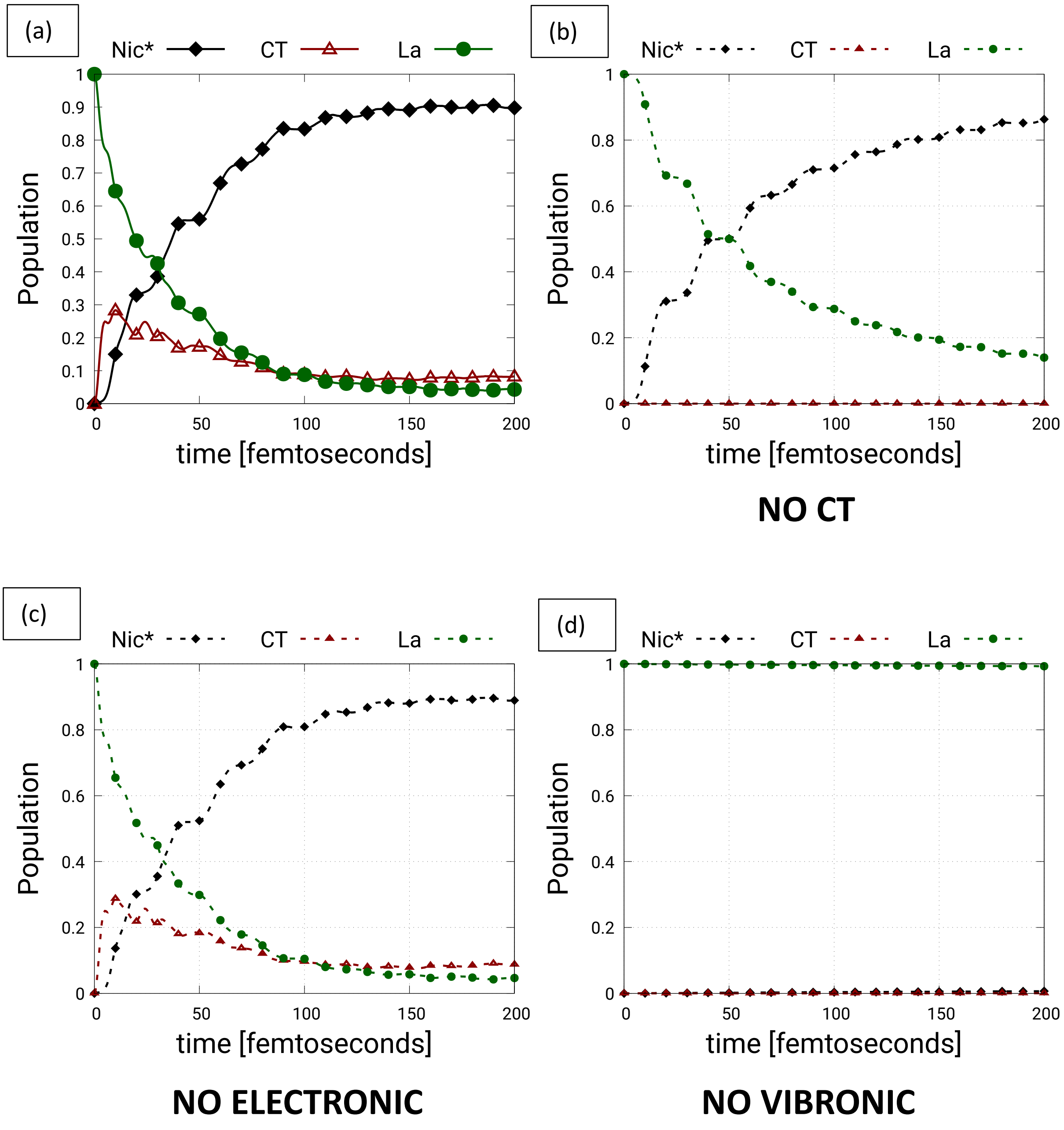

Suppl. Fig. 13.16: Caption same as Suppl. Fig. 13.1

Cluster C2
Structure 05

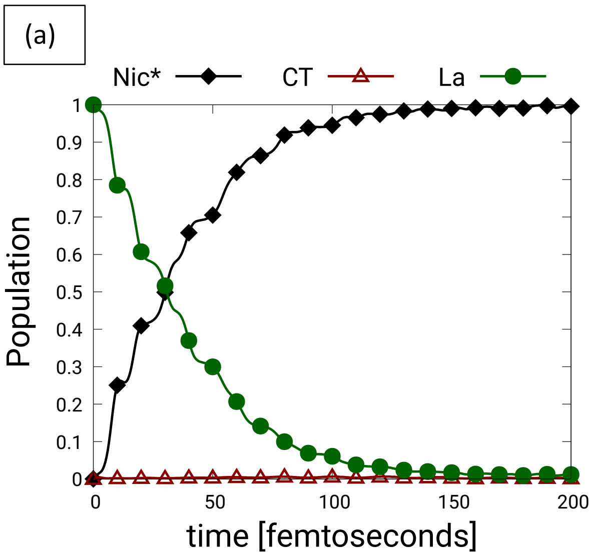

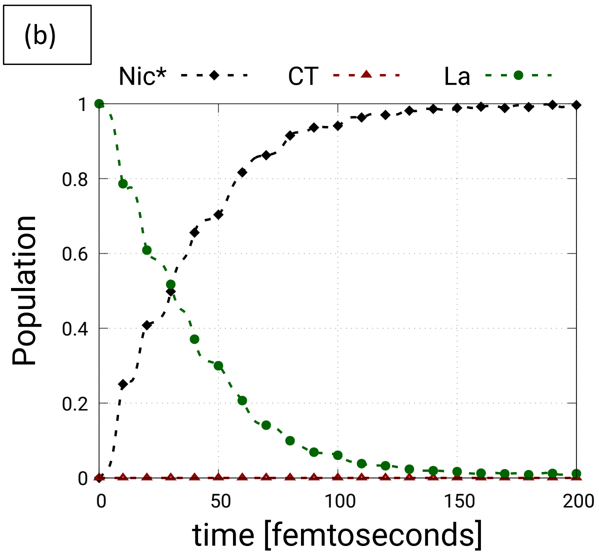

NO CT

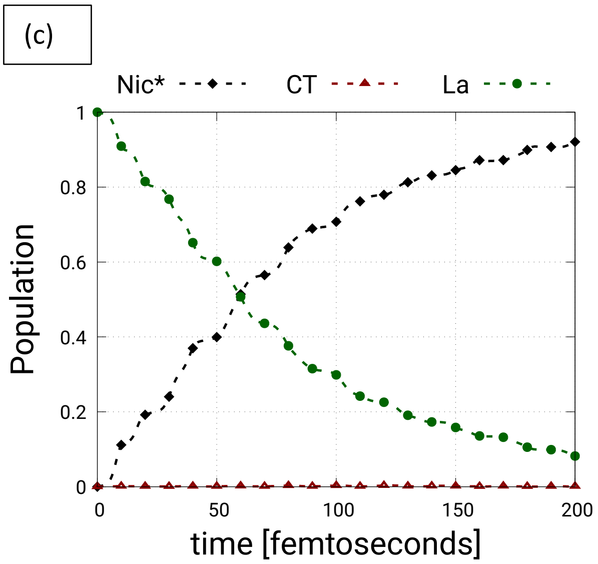

NO ELECTRONIC

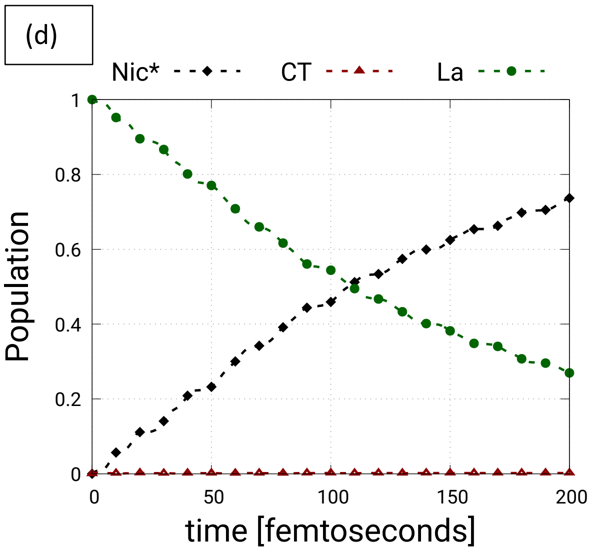

NO VIBRONIC

Suppl. Fig. 13.17: Caption same as Suppl. Fig. 13.1

Cluster C2
Structure 06

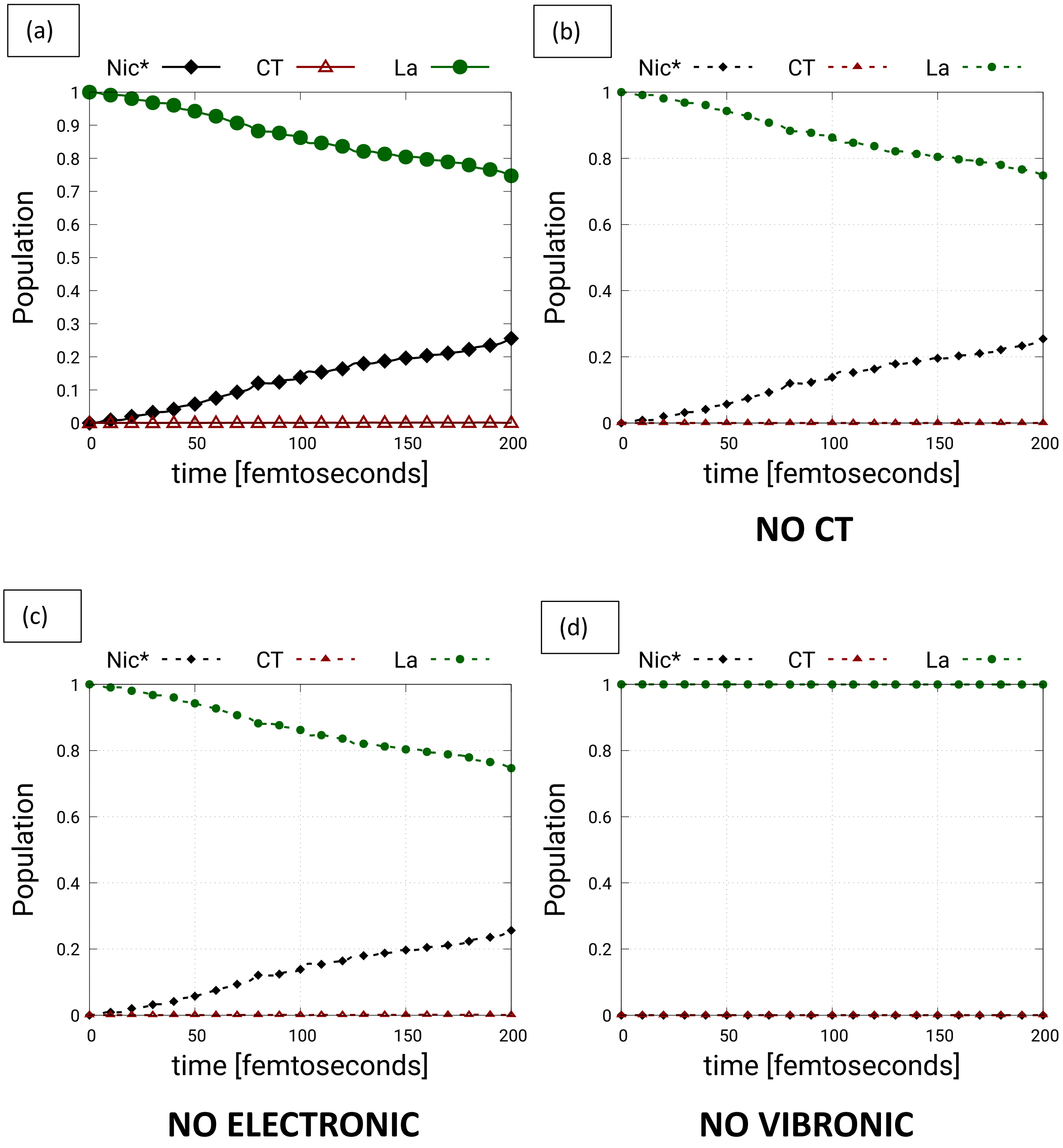

Suppl. Fig. 13.18: Caption same as Suppl. Fig. 13.1

Suppl. Note. 11 Selective effect of removing low-frequency modes on wavepacket dynamics

CLUSTER C0

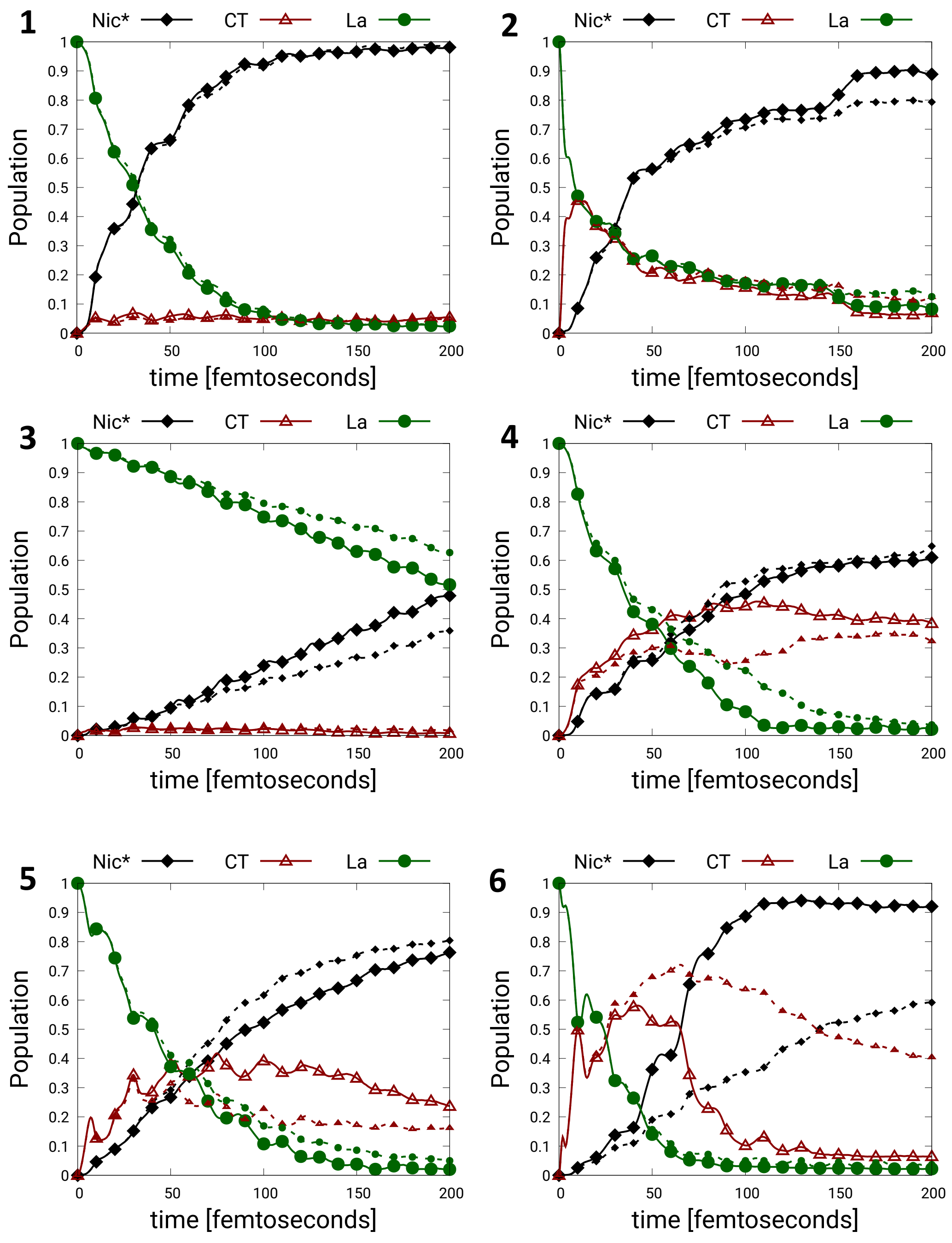

Suppl. Fig. 14: Solid Line: CT-mediated transfer same as Suppl. Fig. 10 including all couplings. Dashed line: CT mediated transfer after removing low frequency modes. Significantly slowed down population transfer is observed for Struct 06, where CT has an active doorway role in ultrafast EET dynamics.

Suppl. Note. 12 Photoactive normal modes responsible for EET transfer

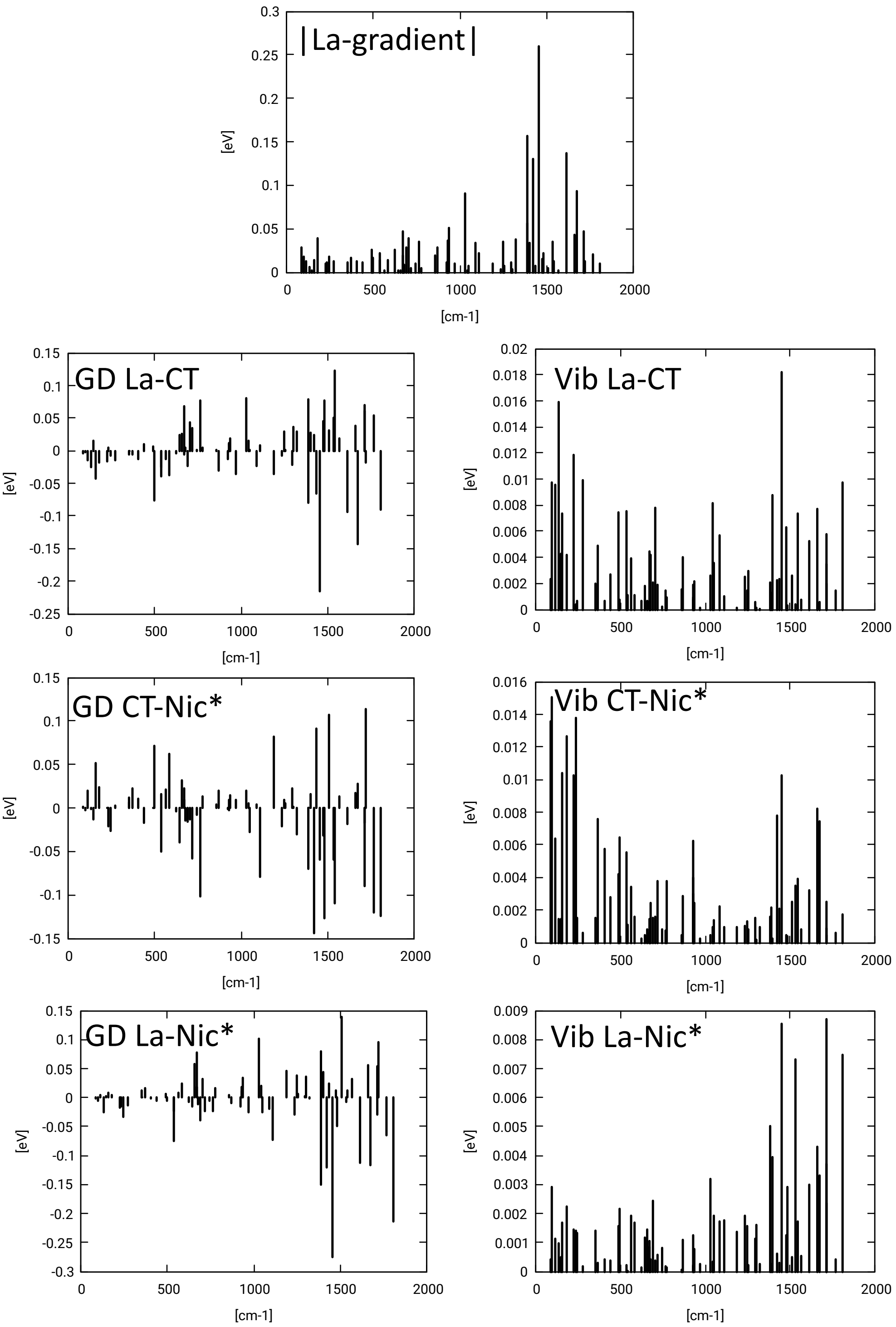

Suppl. Fig. 15: The photoexcited modes responsible for EET for a representative dynamic (Cluster C0 / Structure 06). The absolute value of gradient of La state along the modes , along with interstate gradient difference (GD) and absolute value of vibronic coupling (Vib) along the modes.

Suppl. Note. 13 Branching-space for CT-mediated and direct mechanism

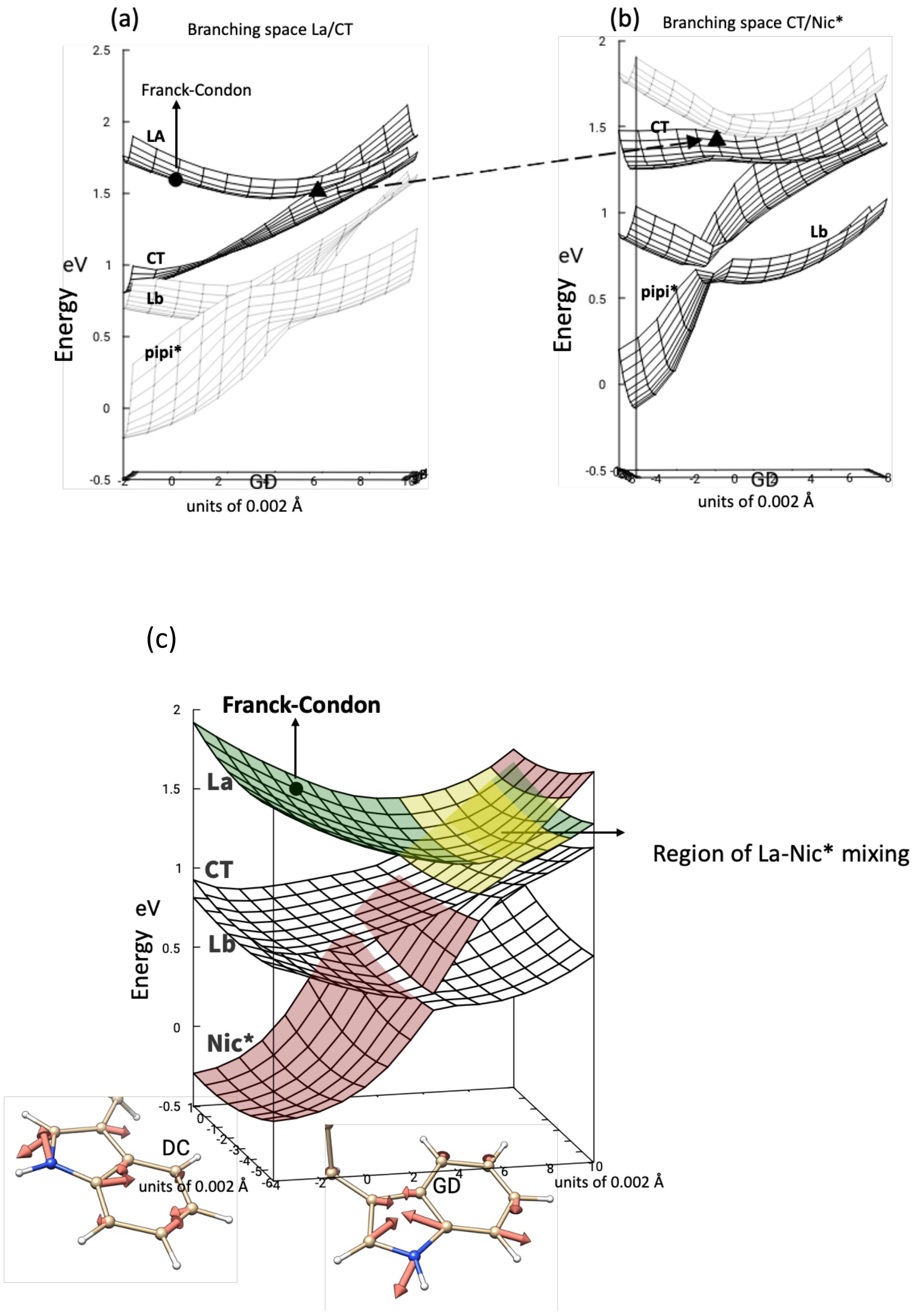

Suppl. Fig. 16: Branching space for (a,b) CT-mediated and (c) Direct EET mechanism showing the conical intersections involving relevant adiabatic states. The branching plane is a 2D PES along gradient difference (GD) and derivative coupling (DC) coordinates in units of Angstroms. These vectors have been computed at the Franck-Condon geometry in the present figures.

Suppl. Note. 14 Wavepacket dynamics on frozen solute in solvent ensemble

14.1 Generation of solvent ensemble

To disentangle the effect of solvent arrangement on the electronic-states and subsequent EET process, an equilibrated solvent ensemble around a fixed solute structure was created. The structure from C0/Struct C6 as labelled in Suppl. Table 6 was taken.

Since the velocities from REMD are not saved, a re-equilibrarion was done by heating the system from 0 to 300K in steps of 10K with 10ps dynamics in each step. This were done with restraints of 200 kcal/(mol·Å^2) to keep the solute fixed. After this, a MD at 300K and NPT for 100ps was done to equilibrate at constant pressure and temperature. Post equilibration, the production MD was run at the same NPT conditions for 200ps. Snapshots were saved every 2ps to obtain decorrelated solvent arrangements around the frozen solute.

A similar QM/MM setup comprising of a spherical droplet as reported in Section 4 was created. The previously optimized solute structure at QM/MM level was re-inserted inside every droplet. To obtain the LVC parameters vertical transition energies at XMS-CASPT2 |4,4| level were computed for every solvent snapshot and diabatized through a solvent-specific rotation matrix $\mathbf{D}_i$ for the i-th solvent snapshot as described in Section 7 to give energies and electronic couplings of solvent-specific LE/CT state.

The solvent specific diabatic states for every snapshot were taken as the reference states for ML-MCTDH dynamics amongst with population transfer was monitored. To obtain vibronic coupling terms, a wavefunction overlap method similar to Section 7 was employed. The overlap matrix $\mathbf{S}_i$ between the adiabatic states at Franck-Condon of **i-th** solvent snapshot $\psi[Q_0]_i$ and the previously computed adiabatic states at displaced geometries were utilized to transform the vibronic Hamiltonian matrix to solvent specific adiabatic-basis. This was then transformed to solvent-specific diabatic matrix through the rotation matrix $\mathbf{D}_i$. This procedure gives solvent specific vibronic-coupling terms along the normal modes for every snapshots of the ensemble.

14.2 Wavepacket dynamics on solvent ensemble in absence of CT-state

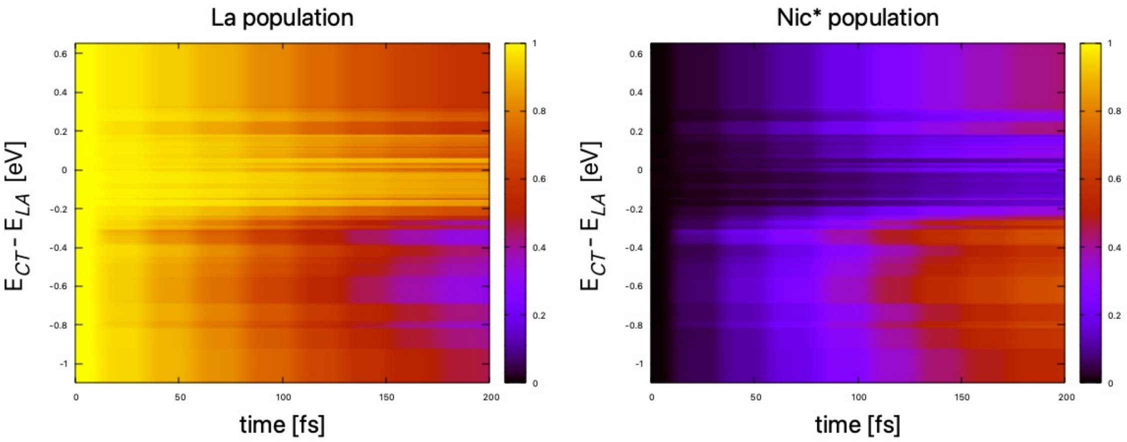

Suppl. Fig. 17: ML-MCTDH dynamics on solvent ensemble around frozen structure from most populated cluster without the CT-state.

Suppl. Note. 15 Coherences between the electronic states along EET dynamics for NADH

We focused on the two cases C0.06 (CT mediated) and C2.05 (direct transfer) investigated respectively in panels 2d and 2f of Figure 2 in the main text. The effect on the EET dynamics of the presence of the CT or of the constant (electronic coupling) and linear (vibronic coupling) terms among all the electronic states was already demonstrated in Figures S13.6 and Suppl. Fig. 13.17 of the SI.

Here we present the coherences for above two cases. We computed the quantum electronic coherences, i.e. the elements of $|La\rangle\langle CT|$, $|La\rangle\langle Nic^*|$, $|CT\rangle\langle Nic^*|$, and namely

$\langle\Psi(t)|La\rangle|\langle CT|\Psi(t)\rangle$, $\langle\Psi(t)|La\rangle|\langle Nic^*|\Psi(t)\rangle$, $\langle\Psi(t)|CT\rangle|\langle Nic^*|\Psi(t)\rangle$. (the other off-diagonal terms of the density matrix are the complex conjugate of these ones). They are represented by complex values and therefore we report both their absolute values and their real and imaginary components. It has been shown that the time dependence of the coherences χ_{ij} between states *i* and *j* is affected by several physical factors[14–16], including the product of the populations of the two states p_i and p_j (a coherence is trivially zero if one of the two states has zero population) and the overlap of the wavepackets components running of their surfaces. In order to disentangle these effects, like proposed in reference [14], we also report normalized coherences defined as $|\chi_{ij}|/\sqrt{p_i p_j}$.

It is interesting to notice that normalized coherences are always larger than true coherences, showing that part of their small values are simply due to the depopulation of one of the two states (the population contribution is largest when $p_i = p_j = 0.5$. On the other side, coherences and normalized coherences show the same recurrence times and this finding supports the interpretation that these recurrence depend on the vibrational coherent motion.

We computed the coherences both including all couplings or selectively switching off some of them (analogously as what done in Section 10 of this SI). Here we further added an additional test, obtained by setting to zero all diagonal gradients. The idea was to maintain the WP at rest in the nuclear coordinate space to check if this helps disentangling vibrational and electronic effects.

The comparative analysis of the results for the two snapshots indicate that the role of the tuning modes is essential for facilitating EET. If we switch them off (labelled λ_{ii}, 5th column in Figures S18 and S19), we observe high frequency oscillations in the coherences proportional to the electronic energy gaps due to the purely electronic coupling E_{ij}^0. Despite the coherences having sizeable values, no substantial population transfer takes place. On these grounds we can conclude that, despite the conformational heterogeneity of NADH, the EET is an intrinsically coherent process governed by coherently oscillating wave packets on the potential energy surfaces of the involved electronic states which tune the energy gaps and drive the EET unidirectionally from the donor to the acceptor. Such coherent EET cannot be described by incoherent mechanisms such a Förster theory

Cluster C0, Structure 06

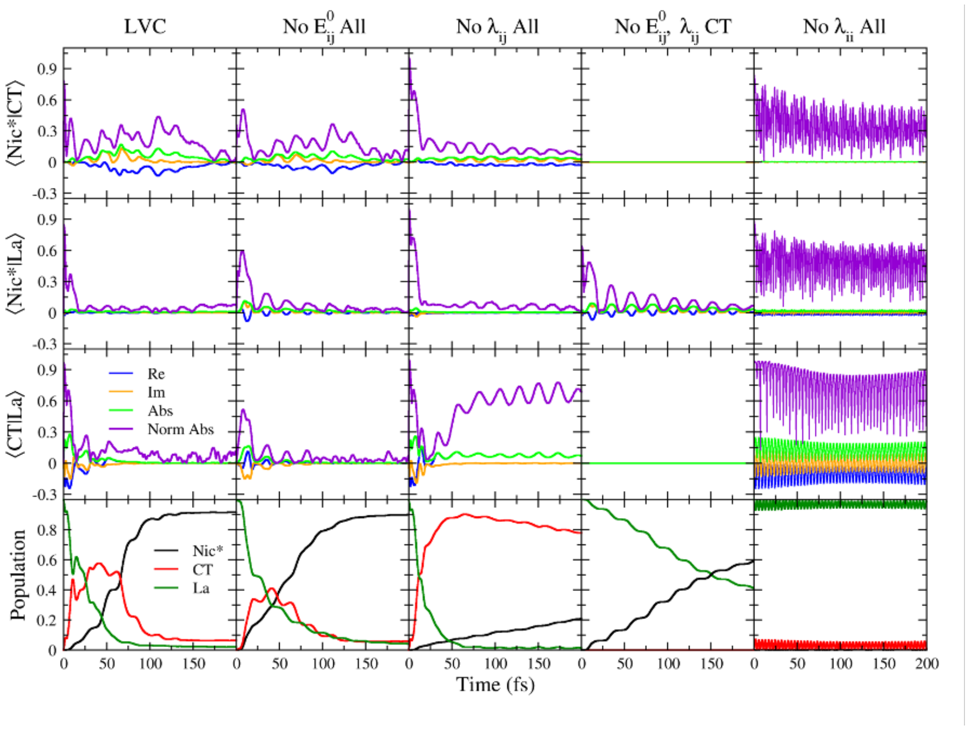

Suppl. Fig. 18: Coherences in EET dynamics involving CT state as an intermediary.

Cluster C2, Structure 05

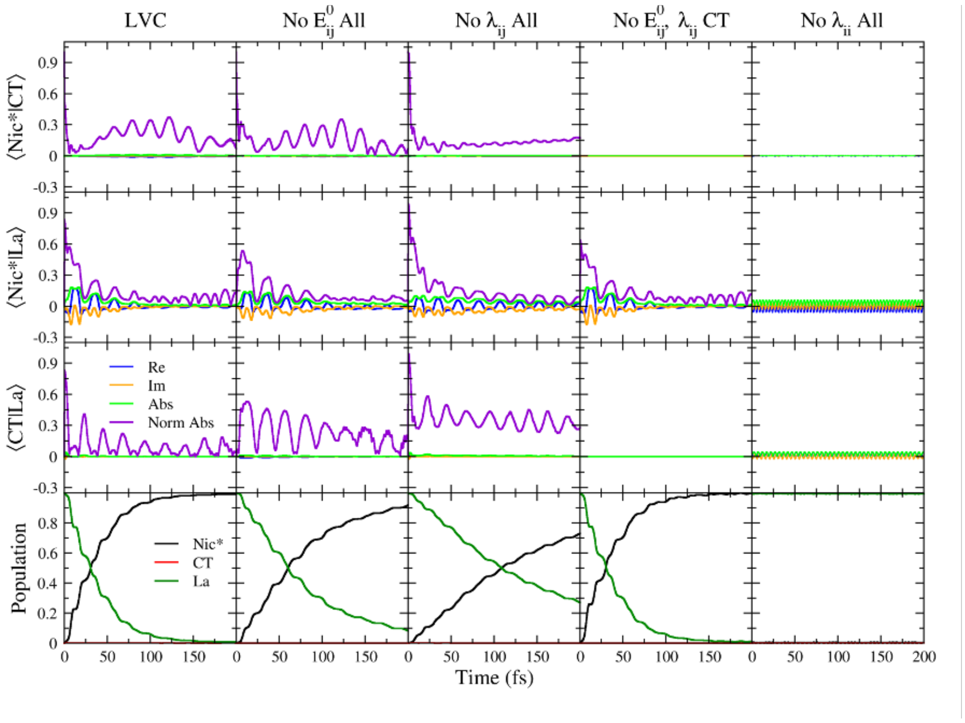

Suppl. Fig. 19: Coherences in EET dynamics involving direct population transfer from La to Nic*

Supplementary References

(1) Heiner, Z.; Roland, T.; Leonard, J.; Haacke, S.; Groma, G. I. Kinetics of Light-Induced Intramolecular Energy Transfer in Different Conformational States of NADH. *J. Phys. Chem. B* **2017**, *121* (34), 8037–8045. https://doi.org/10.1021/acs.jpcb.7b04753.

(2) Cao, S.; Li, H.; Liu, Y.; Zhang, M.; Wang, M.; Zhou, Z.; Chen, J.; Zhang, S.; Xu, J.; Knutson, J. R. Femtosecond Fluorescence Spectra of NADH in Solution: Ultrafast Solvation Dynamics. *The Journal of Physical Chemistry B* **2020**, *124* (5), 771–776. https://doi.org/10.1021/acs.jpcb.9b10656.

(3) Cadena-Caicedo, A.; Gonzalez-Cano, B.; López-Arteaga, R.; Esturau-Escofet, N.; Peon, J. Ultrafast Fluorescence Signals from β-Dihydronicotinamide Adenine Dinucleotide: Resonant Energy Transfer in the Folded and Unfolded Forms. *The Journal of Physical Chemistry B* **2020**, *124* (3), 519–530. https://doi.org/10.1021/acs.jpcb.9b10012.

(4) Reza, M. M.; Durán-Hernández, J.; González-Cano, B.; Jara-Cortés, J.; López-Arteaga, R.; Cadena-Caicedo, A.; Muñoz-Rugeles, L.; Hernández-Trujillo, J.; Peon, J. Primary Photophysics of Nicotinamide Chromophores in Their Oxidized and Reduced Forms. *J. Phys. Chem. B* **2023**, *127* (39), 8432–8445. https://doi.org/10.1021/acs.jpcb.3c03246.

(5) *Handbook of Biochemistry and Molecular Biology*, 3rd ed.; Fasman, G. D., Ed.; CRC Press: London, England, 2021. https://doi.org/10.1201/9780429264214.

(6) McComb, R. B.; Bond, L. W.; Burnett, R. W.; Keech, R. C.; Bowers, G. N., Jr. Determination of the Molar Absorptivity of NADH. *Clinical Chemistry* **1976**, *22* (2), 141–150. https://doi.org/10.1093/clinchem/22.2.141.

(7) Osik, N. A.; Zelentsova, E. A.; Sharshov, K. A.; Tsentalovich, Y. P. Nicotinamide Adenine Dinucleotide Reduced (NADH) Is a Natural UV Filter of Certain Bird Lens. *Scientific Reports* **2022**, *12* (1), 16850. https://doi.org/10.1038/s41598-022-21139-x.

(8) Hayatshahi, H. S.; Henriksen, N. M.; Cheatham, T. E. Consensus Conformations of Dinucleoside Monophosphates Described with Well-Converged Molecular Dynamics Simulations. *Journal of Chemical Theory and Computation* **2018**, *14* (3), 1456–1470. https://doi.org/10.1021/acs.jctc.7b00581.

(9) Pavelites, J. J.; Gao, J.; Bash, P. A.; Mackerell Jr., A. D. A Molecular Mechanics Force Field for NAD+ NADH, and the Pyrophosphate Groups of Nucleotides. *Journal of Computational Chemistry* **1997**, *18* (2), 221–239. https://doi.org/10.1002/(SICI)1096-987X(19970130)18:2<221::AID-JCC7>3.0.CO;2-X.

(10) Walker, R. C.; de Souza, M. M.; Mercer, I. P.; Gould, I. R.; Klug, D. R. Large and Fast Relaxations inside a Protein: Calculation and Measurement of Reorganization Energies in Alcohol Dehydrogenase. *J. Phys. Chem. B* **2002**, *106* (44), 11658–11665. https://doi.org/10.1021/jp0261814.

(11) Ester, M.; Kriegel, H.-P.; Sander, J.; Xu, X. A Density-Based Algorithm for Discovering Clusters in Large Spatial Databases with Noise. In *Proceedings of the Second International Conference on Knowledge Discovery and Data Mining*; KDD'96; AAAI Press: Portland, Oregon, 1996; pp 226–231.

(12) Hsu, C.-P.; You, Z.-Q.; Chen, H.-C. Characterization of the Short-Range Couplings in Excitation Energy Transfer. *J. Phys. Chem. C* **2008**, *112* (4), 1204–1212. https://doi.org/10.1021/jp076512i.

(13) Azumi, T.; Matsuzaki, K. WHAT DOES THE TERM "VIBRONIC COUPLING" MEAN? *Photochemistry and Photobiology* **1977**, *25* (3), 315–326. https://doi.org/10.1111/j.1751-1097.1977.tb06918.x.

(14) Matselyukh, D. T.; Despré, V.; Golubev, N. V.; Kuleff, A. I.; Wörner, H. J. Decoherence and Revival in Attosecond Charge Migration Driven by Non-Adiabatic Dynamics. *Nature Physics* **2022**, *18* (10), 1206–1213. https://doi.org/10.1038/s41567-022-01690-0.

(15) Vacher, M.; Bearpark, M. J.; Robb, M. A.; Malhado, J. P. Electron Dynamics upon Ionization of Polyatomic Molecules: Coupling to Quantum Nuclear Motion and Decoherence. *Phys. Rev. Lett.* **2017**, *118* (8), 083001. https://doi.org/10.1103/PhysRevLett.118.083001.

(16) Fiete, G. A.; Heller, E. J. Semiclassical Theory of Coherence and Decoherence. *Phys. Rev. A* **2003**, *68* (2), 022112. https://doi.org/10.1103/PhysRevA.68.022112.
